# Highly Accurate and Robust Constraint-Based Orbital-Optimized Core Excitations

**DOI:** 10.1021/acs.jpca.4c04139

**Published:** 2024-11-04

**Authors:** Yannick Lemke, Jörg Kussmann, Christian Ochsenfeld

**Affiliations:** † Chair of Theoretical Chemistry, Department of Chemistry, Ludwig-Maximilians-Universität München, Butenandtstr. 5-13, Munich D-81377, Germany; ‡ Max-Planck-Institute for Solid State Research, Heisenbergstr. 1, Stuttgart D-70569, Germany

## Abstract

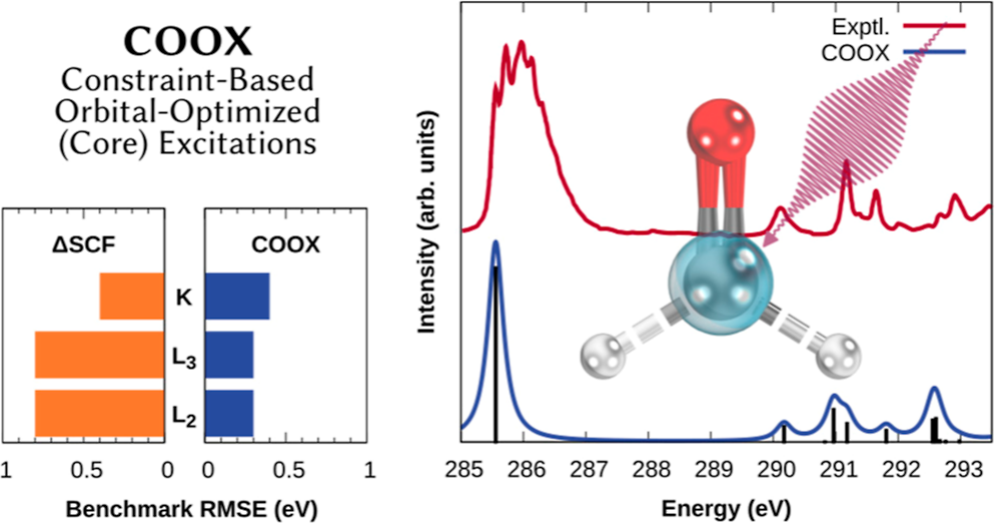

We adapt our recently developed constraint-based orbital-optimized excited-state method (COOX) for the computation of core excitations. COOX is a constrained density functional theory (cDFT) approach based on excitation amplitudes from linear-response time-dependent DFT (LR-TDDFT), and has been shown to provide accurate excitation energies and excited-state properties for valence excitations within a spin-restricted formalism. To extend COOX to core-excited states, we introduce a spin-unrestricted variant which allows us to obtain orbital-optimized core excitations with a single constraint. Using a triplet purification scheme in combination with the constrained unrestricted Hartree–Fock formalism, scalar-relativistic zero-order regular approximation corrections, and a semiempirical treatment of spin–orbit coupling, COOX is shown to produce highly accurate results for K- and L-edge excitations of second- and third-period atoms with subelectronvolt errors despite being based on LR-TDDFT, for which core excitations pose a well-known challenge. L- and M-edge excitations of heavier atoms up to uranium are also computationally feasible and numerically stable, but may require more advanced treatment of relativistic effects. Furthermore, COOX is shown to perform on par with or better than the popular ΔSCF approach while exhibiting more robust convergence, highlighting it as a promising tool for inexpensive and accurate simulations of X-ray absorption spectra.

## Introduction

The elucidation of electronic structure by means of X-ray spectroscopy is becoming increasingly popular in the toolbox of experimental chemistry and physics. Following ongoing advancements of synchrotron radiation sources, spectra can be obtained with ever increasing fidelity, leading X-ray spectroscopy to be coined “an indispensable tool in materials and life sciences.”^1^ The accurate and cost-effective quantum-chemical simulation of such core-excited states is thus highly desirable, both to aid the interpretation of experimental spectra and to help guide the development of new materials, and has subsequently received heightened attention in recent years.^1,2^ Linear-response time-dependent density functional theory (LR-TDDFT),^3,4^ often regarded as the current-day workhorse of electronic structure methods for photochemical applications,^5−7^ struggles with this task considerably: with excitation energies in the range of ∼100 eV to 100 keV, the corresponding wavelengths become short enough to bring into question the validity of the underlying electric dipole approximation. For many light atoms, the dipole contribution is found to still be dominating due to the small spatial extents of the probed core orbitals, thus this approximation appears to not (yet) be problematic;^8^ however, studies on K-edge excitations of transition metal compounds have found non-negligible quadrupole contributions to the predicted intensities which substantially shape the computed spectra.^9,10^ Nevertheless, the accuracy of LR-TDDFT for core excitations leaves much to be desired: excitation energies are typically underestimated by a substantial margin and require the manual alignment of computed spectra with experiment,^11^ limiting the applicability of LR-TDDFT for ab initio simulations of X-ray absorption spectra if accurate excitation energies are of interest (though it should be noted that the *precision* of LR-TDDFT, i.e., relative energies between core-excited states, is found to be much better^12^). This shortcoming is usually attributed to the pervasive self-interaction error in density functional theory^13−16^ and can be understood—similarly to the so-called “electron-transfer self-interaction error” occurring in the description of charge-transfer states by means of LR-TDDFT^17,18^—as a consequence of lacking orbital relaxation within the linear-response formalism.^1^ Inclusion of exact exchange (EXX) in the exchange–correlation (xc) functional can recover a partial description of these effects, and the magnitude of the required shift thus greatly depends on the respective xc functionals used. A number of short-range corrected functionals have been developed specifically to overcome these deficiencies,^11,19−21^ though questions regarding their general-purpose applicability outside of core excitations remain. Moreover, functionals optimized on ionization potentials and Rydberg states such as the “Quantum Theory Project” (QTP) functionals of Bartlett and co-workers^22,23^ have been shown to perform comparatively well for the computation of near-edge X-ray absorption spectra through LR-TDDFT.^24^

Going beyond the realm of time-dependent DFT, higher-level methods such as equation-of-motion coupled-cluster^25,26^ and algebraic diagrammatic construction to second order (ADC(2))^27^ also routinely require manual alignment of computed spectra, though the shifts are typically one or 2 orders of magnitude smaller than those required for LR-TDDFT.^27−29^ In particular, the extended core–valence-separated ADC(2) approach was found to provide both good accuracy and precision,^12^ though combined with their rather steep computational scaling, the applicability of such methods to larger molecular clusters or solids is also somewhat limited.

In recent years, there has been renewed interest in orbital-optimized methods, which compute excited states on a state-by-state basis through subsequent self-consistent field (SCF) calculations modified in such a way that the excited state of interest is obtained. Among these, the ΔSCF method^30−32^ is certainly the most well-known, and there has been a recent resurgence of applications employing ΔSCF, e.g., in the context of nonadiabatic molecular dynamics.^33,34^ While such orbital-optimized approaches lack a formal theoretical justification since there exists no Hohenberg–Kohn theorem^35^ for excited states^36^ (besides other points of discussion regarding the validity of time-dependent DFT^37−39^), local excitation energies can often be obtained with similar levels of accuracy as for LR-TDDFT^40^ given physically motivated excitation patterns. Furthermore, orbital-optimized methods can successfully overcome some of the shortcomings of LR-TDDFT, such as the description of double excitations (which LR-TDDFT is entirely incapable of),^41,42^ Rydberg states,^43,44^ charge-transfer states,^17,18,45−48^ and, of particular relevance for this work, core-excited states.^49,50^ Despite its successes, however, a number of computational hurdles remain for the ΔSCF method in particular, most notably the phenomenon of *variational collapse* to the ground state due to the violation of the aufbau principle. A number of approaches have been developed to tackle this issue,^51−54^ though convergence is still not always straightforward, as we shall demonstrate in this work. Furthermore, the problem of spin-contamination must be addressed through purification schemes^55^ or alternatively through the application of restricted open-shell approaches^56,57^ with squared-gradient minimization.^58,59^

Constrained DFT (cDFT)^60−63^ represents an alternative approach to the rather manual generation of excited states encountered in ΔSCF. Instead of user-defined transitions, excited states are generated through the addition of a constraint potential to the effective Hamiltonian. Originally, mostly spatial potentials were used, which lend themselves nicely to the generation of charge-transfer or spin-polarized states.^61−63^ Local Frenkel excitations or core excitations, on the other hand, are difficult to describe using fragment-based spatial constraints, and recent attention toward these problems has resulted in the development of further cDFT variants, namely the “excited cDFT” (x-cDFT) method of Ramos and Pavanello,^64^ the transition-based cDFT approach (t-cDFT) of Stella et al.,^65^ and our recently developed constraint-based orbital-optimized excited state method (COOX)^66^ designed in part to specifically address some of the shortcomings of x-cDFT and t-cDFT. In this paper, it is our aim to extend COOX to the description of core-excited states.

Our manuscript is thus structured as follows: the Theory section provides a brief overview of the working equations of cDFT and the definition of the constraint potential for COOX, followed by the modification of COOX for the description of core excitations. In the Computational Details section, we describe our computational setup, including the description of relativistic effects, used throughout this work. In the Results and Discussion section, we benchmark COOX extensively next to ΔSCF and LR-TDDFT compared to experimental data on K-, L-, and M-edge excitation energies, spectra, and optimized geometries. Finally, we give some concluding remarks and an outlook on remaining challenges and potential future directions.

## Theory

### Constrained DFT

The basic idea of constrained DFT is to generate orbital-optimized excited states by imposing an additional constraint potential *W*_c_ upon the electron density.^60−62^ The corresponding Lagrange functional takes the form
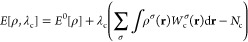
1
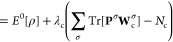
2where *E*^0^[ρ] is the usual Kohn–Sham energy functional, λ_c_ is the Lagrange multiplier corresponding to the constraint potential *W*_c_ with target value *N*_c_, and σ = {α, β} denotes the electron spin. In eq 2, we introduce the one-particle (spin-)density matrix **P** as well as a matrix representation for the constraint potential in the atomic orbital basis (denoted by indices μ, ν, ...)
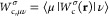
3

The energy of the system is obtained in a self-consistent fashion by requiring eq 1 to become stationary with respect to both the density ρ and the Lagrange multiplier λ_c_; more precisely, the desired stationary point is a minimum with respect to ρ and a maximum with respect to λ_c_^63^
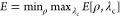
4

For further details regarding the foundations of cDFT, we refer the reader to ref (63).

### Constraint-Based Orbital-Optimized Excitations

In this work, we use our recently developed constraint-based orbital-optimized excited-state method (COOX)^66^ to variationally evaluate excited states. Within COOX, we require the projection of the excited-state density matrix onto the static part of the difference density of a preceding (simplified) LR-TDDFT calculation to be zero, that is
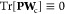
5with
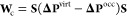
6
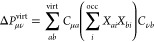
7
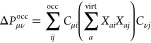
8where **S** is the atomic orbital overlap matrix, **C** are the ground-state molecular orbital coefficients, and **X** are the transition amplitudes obtained from a (simplified) Tamm–Dancoff approximation (TDA) calculation (for analogous expressions using full LR-TDDFT, the reader is referred to the Supporting Information of ref (66)). Mathematically, the constraint can be interpreted as follows: the matrices **SΔP**^virt^**S** and **SΔP**^occ^**S** act as projectors onto the respective virtual and occupied orbital subspaces which comprise the excitation. Therefore, requiring the overall constraint value to be zero enforces that a converged excited-state density **P** contains an equal number of electrons in the subspaces projected onto by **ΔP**^virt^ and **ΔP**^occ^. For local excitations in which the occupied orbital space is predominantly defined by a single orbital, this then leads to the desired single excitation. For instance, consider a single excitation from the highest occupied to the lowest unoccupied molecular orbital (HOMO to LUMO). In this case, the transition amplitudes **X** simplify to a product of Kronecker deltas, , leading to the projectors
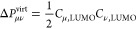
9
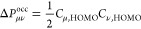
10

Clearly, for the ground state density **P**_0_, we have
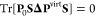
11
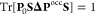
12i.e., two electrons in the **ΔP**^occ^ subspace (note the normalization factor of 1/2) and no electrons in the **ΔP**^virt^ subspace. A single excitation should thus excite one of the two electrons into the **ΔP**^virt^ subspace, whereas the other electron should remain within the **ΔP**^occ^ subspace, i.e., the excited-state density **P** should satisfy
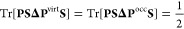
13or equivalently
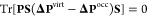
14

Similar to other orbital-optimized methods, COOX-based excited-state wave functions are generally nonorthogonal to the ground state and to each other. The issue of nonorthogonality is an ongoing point of discussion for orbital-optimized methods in general^64,67,68^ since the ground-state reference determinant in KS-DFT does not represent the true wave function and thus, strict orthogonality might not be a requirement. One-electron excited-state properties such as the transition dipole moment between two states |Φ_*I*_⟩ and |Φ_*J*_⟩ are easily attainable using Löwdin’s generalized Slater–Condon rules for the transition density^69^
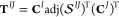
15where **C**^*I*^ and **C**^*J*^ are the occupied MO coefficients of states *I* and *J*
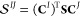
16is the MO overlap matrix, and  denotes the adjugate of . Using these definitions, the wave function overlap is given as
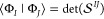
17whereas one-particle operators can be evaluated using
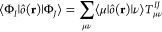
18

### Core Excitations

**Figure 1:**
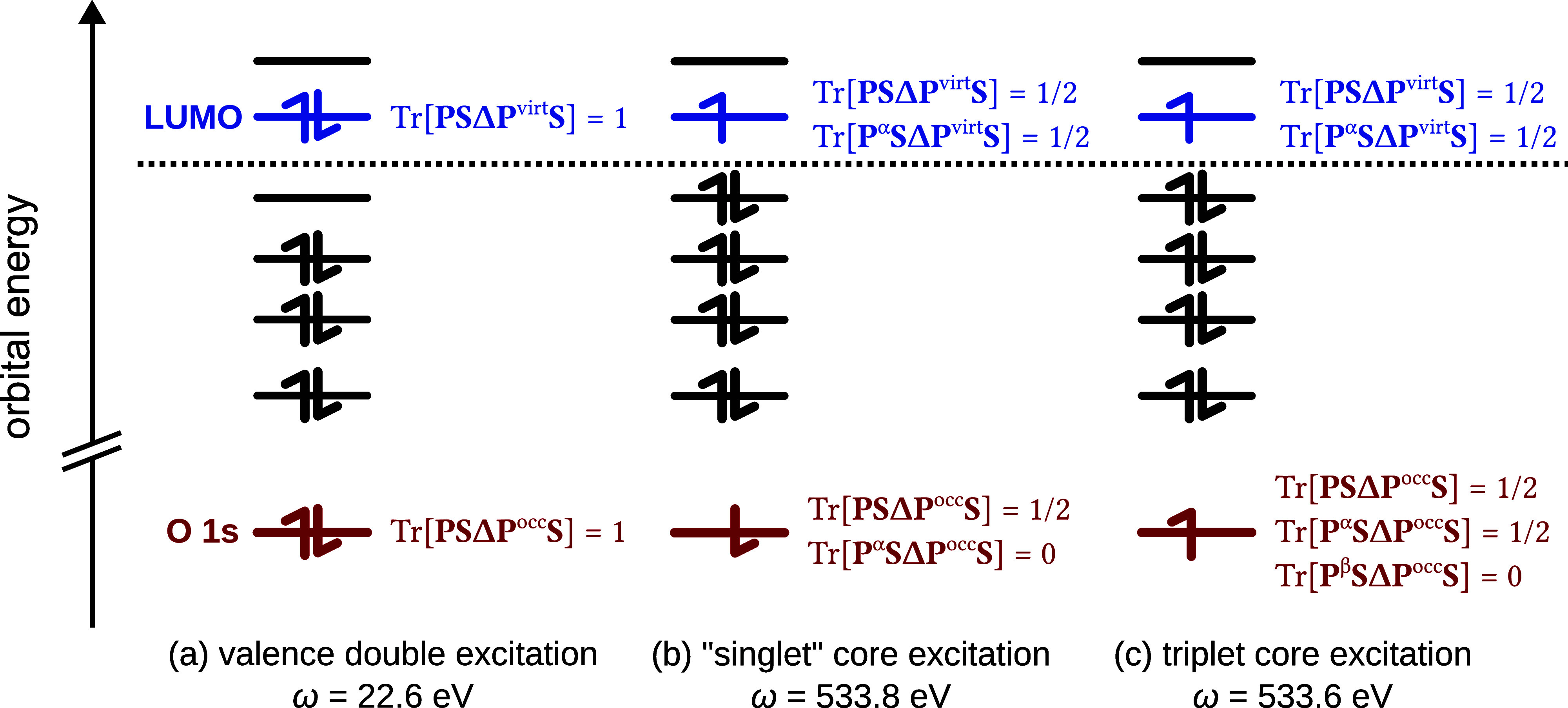
Pictorial representation of (a) a valence double excitation, (b) a spin-contaminated singlet core excitation, and (c) a triplet core excitation from the O 1s orbital of water (excitation energies computed at the COOX/PBE0/aug-pcX-2 level of theory, see Results and Discussion). All excitations satisfy the constraints defined in eqs 6–8, but the excitation energy for the double excitation is considerably lower. Only case (b) satisfies the constraints outlined in eqs 22 and 23, whereas only case (c) satisfies eqs 27–29.

In order to generate orbital-optimized core-excited states with COOX, we need to modify both the potential used within the cDFT-SCF as well as the preceding LR-TDDFT calculation. First, let us consider the LR-TDDFT part: to obtain a core-excited state without requiring the calculation of an unfeasibly large number of roots, we enforce a core-hole by restricting the active occupied space to the core orbital in question. This so-called core–valence separation (CVS) scheme is a commonly used approach and the effect on the accuracy of the resulting excitation energies has been found to be insubstantial.^70,71^ Next, recalling that the COOX constraint potential requires as many electrons to be excited into **ΔP**^virt^ as remain in **ΔP**^occ^, it is clear that an optimized core-excited state would in theory satisfy the constraints outlined in eqs 5–8. However, such a state cannot be obtained through a regular cDFT-SCF as is the case for local excitations. This is because the SCF procedure attempts to find a minimal energy solution that satisfies the imposed constraint, which results in an undesired double-excitation in which two valence electrons are excited into the virtual space and both core electrons remain in the core orbital, thus fulfilling the constraint. A schematic representation of this is shown in Figure 1 for the water molecule: both the core excitation and the valence double excitation satisfy the constraint, but the valence double excitation energy is far lower, thus driving the SCF toward this solution instead of the desired core excitation.

It is therefore necessary to use different types of constraints. In principle, one could split **W**_c_ into two separate constraints, i.e.
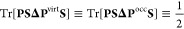
19(note that ), which would allow us to maintain the restricted formalism when combined with Fermi-smearing. However, this approach was found to yield unsatisfactory results with K-edge excitation energies that are about 5–10 eV too high, owing to a spurious excitation of valence electrons within the first few iterations in which the optimization of the cDFT Lagrange multiplier can be numerically unstable, as well as considerable convergence problems for L-edge excitations, and will thus not be discussed in detail. Alternatively, we can employ a spin-polarized ansatz in which we only constrain the spin-up density. The excited-state density of such a spin-polarized state would yield the constraint values
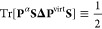
20
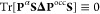
21as is shown in Figure 1b. This relation straightforwardly allows us to combine eqs 20 and 21 in a single constraint as
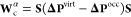
22
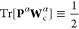
23

It should be noted that this approach has some drawbacks over the simpler restricted approach used for local excitations: first, because the constraint no longer evaluates to zero, the free energy of the constrained system no longer coincides with the expectation value of the unconstrained Hamiltonian; thus, the excited state no longer resembles a free oscillation. While the resemblance of such a free oscillation is by no means a requirement, this means it is not necessarily justified to compute the Pulay correction to analytic gradients from an unperturbed Kohn–Sham matrix, as we shall discuss at a later point in this work. Second, the resulting states are severely spin-contaminated with , which reflects a well-known problem within ΔSCF and x-cDFT. To overcome this issue, we make use of the approximate spin-projection (AP) procedure of Yamaguchi et al.^55^ In this procedure, the energy of the singlet state is computed from the energy of the spin-contaminated mixed state and the corresponding triplet state as
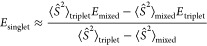
24

To obtain the required triplet state, we need to excite an electron from the spin-down part of **ΔP**^occ^ into the spin-up part of **ΔP**^virt^. Thus, in analogy to eqs 20 and 21, the excited-state density **P** = **P**^α^ + **P**^β^ should satisfy the relations
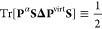
25
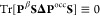
26as shown in Figure 1c, yielding the spin-up and spin-down constraints
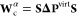
27
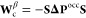
28
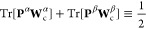
29

The overall spin-purification procedure then reads as follows:

1. Compute a relaxed spin-contaminated singlet excitation (*S* = 0, *N*_α_ = *N*_β_) using the constraints defined in eqs 22 and 23 to obtain *E*_mixed_ and .
2. Compute a relaxed triplet excitation (*S* = 1, *M*_S_ = 1, *N*_α_ = *N*_β_ + 2) using the constraints defined in eqs 27–29 to obtain *E*_triplet_ and .
3. Compute the purified singlet excitation energy according to eq 24.

As is the case in ΔSCF and x-cDFT, spin-contamination of the COOX triplet state is much less prominent than for the singlet state; nevertheless, we have found it beneficial to employ the constrained unrestricted Hartree–Fock formalism of Tsuchimochi and Scuseria^72^ to minimize spin-contamination of the triplet wave function as much as possible.

## Computational Details

All herein presented methods have been implemented into the FermiONs++ program package.^73,74^ Throughout, we use tight thresholds for integral screening (ϑ_int_ = 10^–10^) and SCF convergence determined by rms[**FPS** – **SPF**] (ϑ_SCF_ = 10^–7^). For the computation of K- and L-edge excitations of second- and third-period elements, we use the aug-pcX-2 basis set of Ambroise and Jensen.^75^ For the computation of L- and M-edge excitations of fourth- and fifth-period elements, we employ the aug-cc-pwCVTZ-DK basis set of Peterson and co-workers (using aug-cc-pwCVTZ for lighter elements),^76,77^ and for the computation of L- and M-edge excitations of sixth- and seventh-period elements, we use a combination of the ma-ZORA-def2-TZVPP^78^ and SARC-ZORA-TZVPP basis sets.^79,80^ Numerical quadratures for the xc functional are evaluated through use of the LibXC library^81^ on a gm5 grid,^82^ and the sn-LinK method^83−85^ with a gm4 grid^82^ is used for the computation of EXX. The cDFT Lagrange multiplier is determined through the TOMS748 bracketing algorithm^86^ as implemented in the Boost library with a tight convergence threshold of 10^–10^ for |Tr[**PW**_c_] – *N*_c_|. For ΔSCF calculations, we use the initial maximum overlap method^51,52^ to aid convergence and avoid variational collapse.

TDA calculations are carried out using an iterative Davidson procedure with a convergence criterion of 10^–6^ for the root-mean-square of the residuals. We employ a CVS scheme in which the occupied space is restricted to the core orbital in question, and always include at least two additional roots besides the desired state. COOX constraints are then computed from the amplitudes of the lowest symmetry-allowed TDA transition, i.e., the lowest state with a nonzero transition dipole moment.

For excitations from symmetry-equivalent atoms, the core orbitals are delocalized (e.g., C in C_2_H_6_). As was shown by Hait and Head-Gordon, a delocalized hole leads to considerable underestimation of the excitation energy;^59^ thus, we perform an orbital localization following the ground-state SCF if the core orbital in question belongs to a symmetry-equivalent atom.

Scalar-relativistic effects are accounted for by virtue of the zero-order regular approximation (ZORA)^87,88^ using the effective potential introduced by van Wüllen^89^ evaluated on a g5 grid for second- and third-period elements and a g7 grid for heavier elements. It should be noted that the ZORA Hamiltonian does not necessarily present an ideal choice for core properties compared to more involved methods like the exact two-component method (X2C), however, a full comparison of relativistic schemes exceeds the scope of this publication. For reference, we include excitation energies obtained with the scaled-ZORA variant^90^ in the Supporting Information, and note in passing that the deviations between ZORA and scaled-ZORA are near negligible for most systems studied in this work (on the order of 0.1% of the excitation energy) with the exception of systems containing heavy atoms. To account for spin–orbit coupling, we use the semiempirical method of Hait and Head-Gordon,^59^ which we also describe in more detail in the Supporting Information.

## Results and Discussion

### K-Edge Excitations of Second-Period Atoms

**Figure 2:**
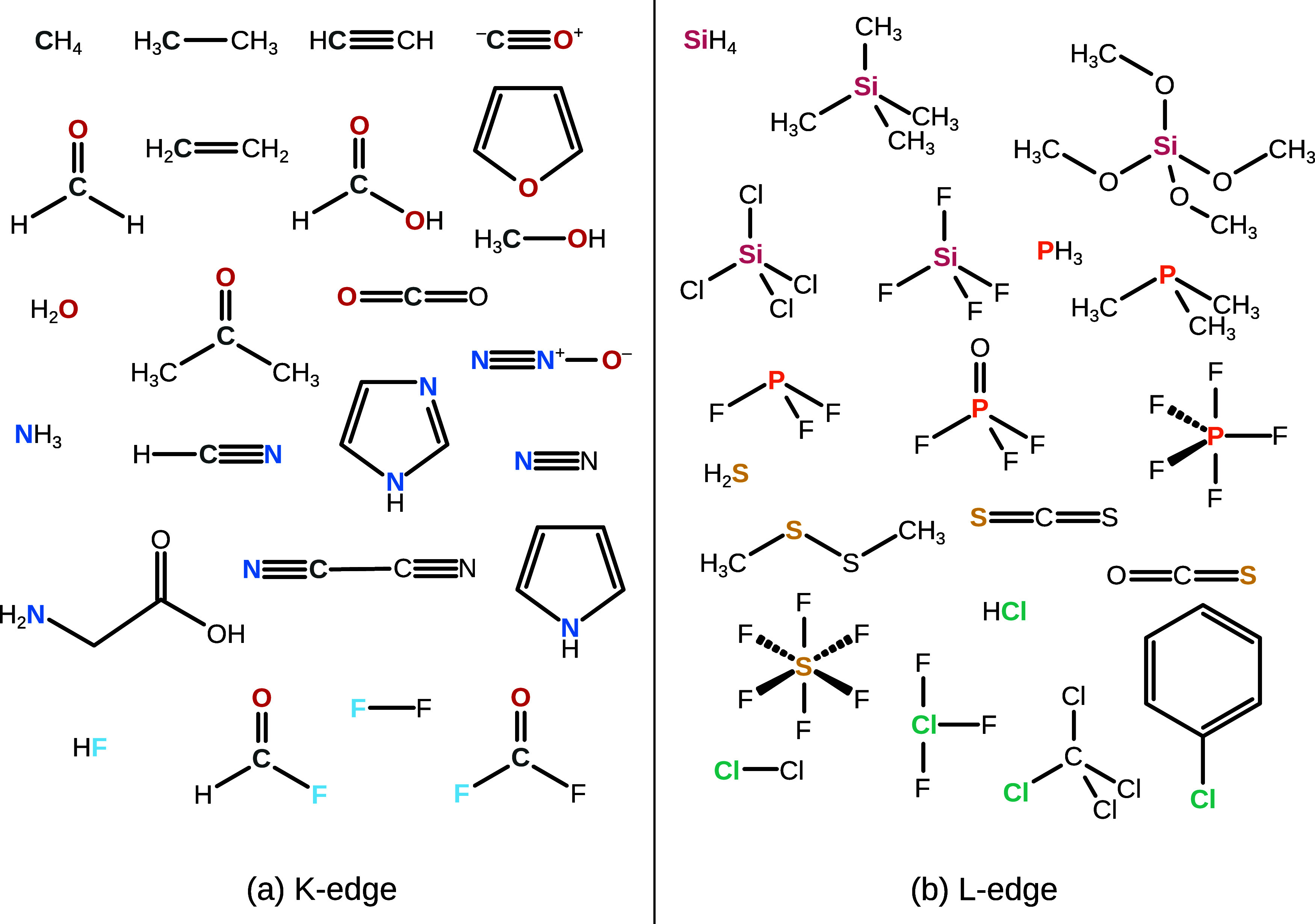
Benchmark systems investigated in this work for (a) K-edge and (b) L-edge excitations. Atoms whose core orbitals are probed are highlighted in bold and with color. See Tables S1 and S5 in the Supporting Information for experimental data. Geometries taken from ref (59).

We first investigate a benchmark of 40 K-edge excitations from second-period atoms that was introduced in ref (59). The molecules in question are illustrated in Figure 2a, and computed excitation energies are referenced against experimental data from refs (91–105) (see Table S1 in the Supporting Information for individual references). As discussed in the Computational Details, we compute the constraint potential for each COOX state from the amplitudes of the lowest symmetry-allowed TDA state, where we restrict the active space of the TDA calculation to the core-orbital in question.

In terms of xc functionals, we chose the generalized-gradient approximation functional of Perdew, Burke, and Ernzerhof (PBE),^106^ its hybrid counterpart PBE0 (often also referred to as PBEh),^107,108^ which adds 25% of exact Hartree–Fock exchange, and the range-separated hybrid ωB97X^109^ functional with 15.77% of short-range EXX and 100% of long-range EXX.

We also include scalar-relativistic corrections in the form of the zero-order regular approximation for ΔSCF and COOX calculations, which leads to K-edge excitation energies that are on average 0.6 eV higher compared to calculations without ZORA. It is worth noting that for the ωB97X functional, the inclusion of ZORA leads to slightly worse results for both ΔSCF and COOX in the sense that we obtain higher errors compared to the experimental data; nevertheless, all calculations presented in the main text were performed with ZORA for the sake of consistency. A comprehensive overview of all K-edge excitation energies with and without regular and scaled ZORA, as well as resulting mean errors (ME), mean absolute errors (MAE), and root mean squared errors (RMSE) metrics, can be found in Tables S1–S3 of the Supporting Information.

**Figure 3:**
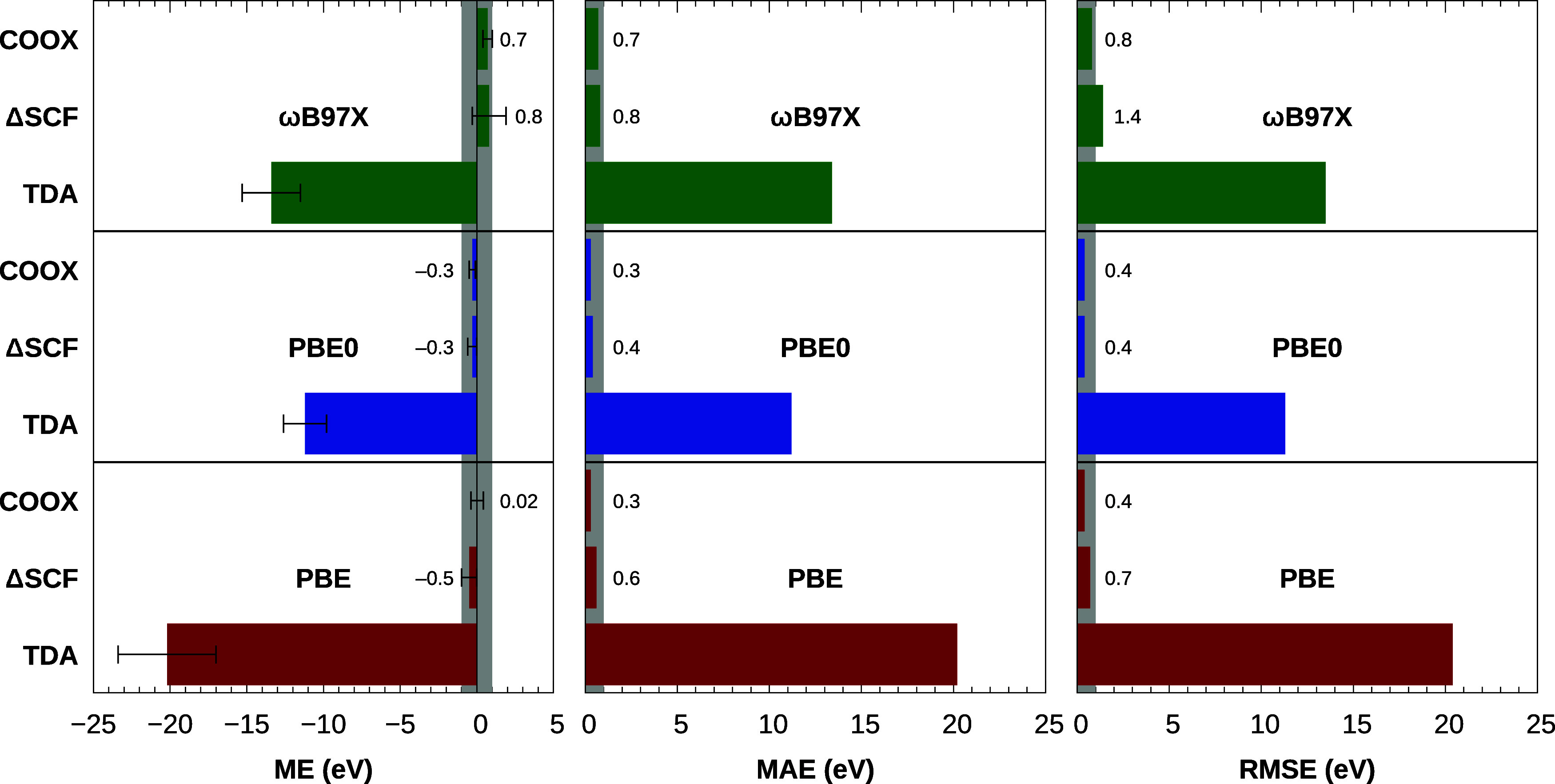
Mean errors with standard deviation (error bars), mean absolute errors, and root mean squared errors (in eV) for K-edge excitation energies of second-period elements in small molecules computed at the TDA, ΔSCF, and COOX levels of theory with the PBE, PBE0 and ωB97X functionals using the aug-pcX-2 basis set and scalar-relativistic ZORA corrections (see Computational Details and Supporting Information for more information). An error region of ±1 eV is shaded in gray.

Figure 3 shows an overview of the ME, MAE, and RMSE for this benchmark computed using TDA, ΔSCF, and COOX. As discussed previously, LR-TDDFT and the TDA are well-known to produce considerable errors for core excitations, necessitating a manual shift to align computed spectra with experimental data. This deficiency is reflected in Figure 3, where we see a systematic underestimation of K-edge excitation energies with mean errors of −20.2 eV for PBE, −11.2 eV for PBE0, and −13.4 eV for ωB97X. Both orbital-optimized methods offer significantly better performance; for both ΔSCF and COOX, mean errors and mean absolute errors are below 1 eV.

The overall best results are obtained for the COOX method with the PBE functional, yielding a near-zero mean error of 0.02 eV, a mean absolute error of 0.3 eV, and a root mean squared error of 0.4 eV. The PBE0 functional matches these results in the MAE and RMSE metrics, while for ωB97X, we observe on average a slight overestimation of the excitation energy with a mean error of +0.8 eV for ΔSCF and +0.7 eV for COOX. Overall, our COOX method is seen to perform on par or in most cases even better than ΔSCF for K-edge excitations as COOX not only produces identical or smaller errors, but also smaller standard deviations than ΔSCF, which is particularly promising due to the stable convergence afforded by the cDFT approach and the “black-box” nature of COOX in comparison to ΔSCF.

**Figure 4:**
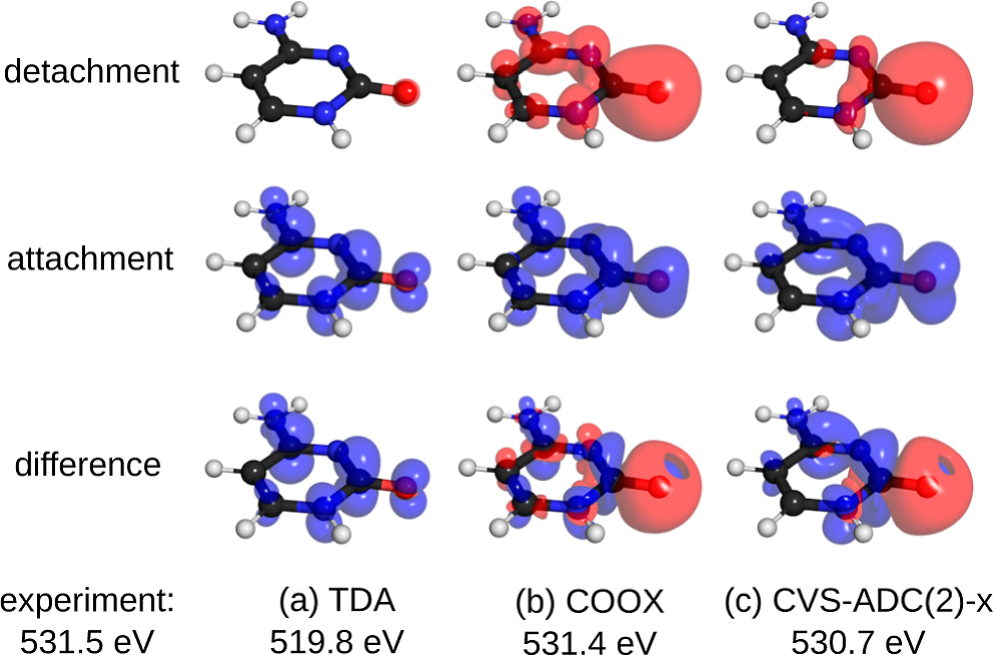
Detachment, attachment, and difference densities for the oxygen K-edge excitation of cytosine computed with the aug-pcX-2 basis set for (a) TDA@PBE0, (b) COOX@PBE0, and (c) CVS-ADC(2)-x. Experimental value from ref (111).

The magnitude of the relaxation of the electronic structure can be seen in Figure 4, where we show the detachment, attachment, and difference densities for the oxygen K-edge excitation of cytosine for TDA and COOX using the PBE0 functional. In addition, results from the core–valence-separated extended algebraic diagrammatic construction theory to second order (CVS-ADC(2)-x)^29^ computed with the Q-Chem 5.1 program package^110^ using the aug-pcX-2 basis set are shown. Evidently, the TDA detachment density is highly localized on the oxygen 1s atom, whereas COOX and CVS-ADC(2)-x correctly capture the relaxation of the electronic structure and thus provide excitation energies which are in far better agreement with experimental values.^111^

The computation of full X-ray absorption spectra by means of COOX is also trivially realized through the inclusion and subsequent COOX optimization of more roots within the TDA calculation. This, we believe, marks a distinct advantage over ΔSCF, where excitations not only need to be manually chosen, but mixed excitation patterns (e.g., 50% 1s → LUMO and 50% 1s → LUMO + 1) are all but inaccessible. Furthermore, computing X-ray absorption spectra through COOX can be done in a highly efficient fashion, as the straightforward definition of the constraint potential allows the cDFT-SCF calculations to be easily distributed across multiple compute nodes once the initial ground-state SCF and TDA excited-state calculations have finished. Some care must be taken in the computation of the transition density matrix due to the nonorthogonality of the obtained Slater determinants; for this purpose, we use the transformation of Bourne-Worster et al.,^68^ though we would like to note that unlike stated in ref (68), calculation of the transition density matrix requires the cofactor matrix of the orbital overlap, i.e., the transposed adjugate as shown in eq 15, and not the adjugate itself. It should also be noted that the Slater determinant describing the fictitious noninteracting Kohn–Sham system does not coincide with the true wave function, thus the transition dipole moment and derived oscillator strengths are not rigorously defined. However, computing the transition dipole moment from the Kohn–Sham determinants is a commonly used practice in orbital-optimized methods^51,59,68^ and has been found to provide reasonable oscillator strengths.

**Figure 5:**
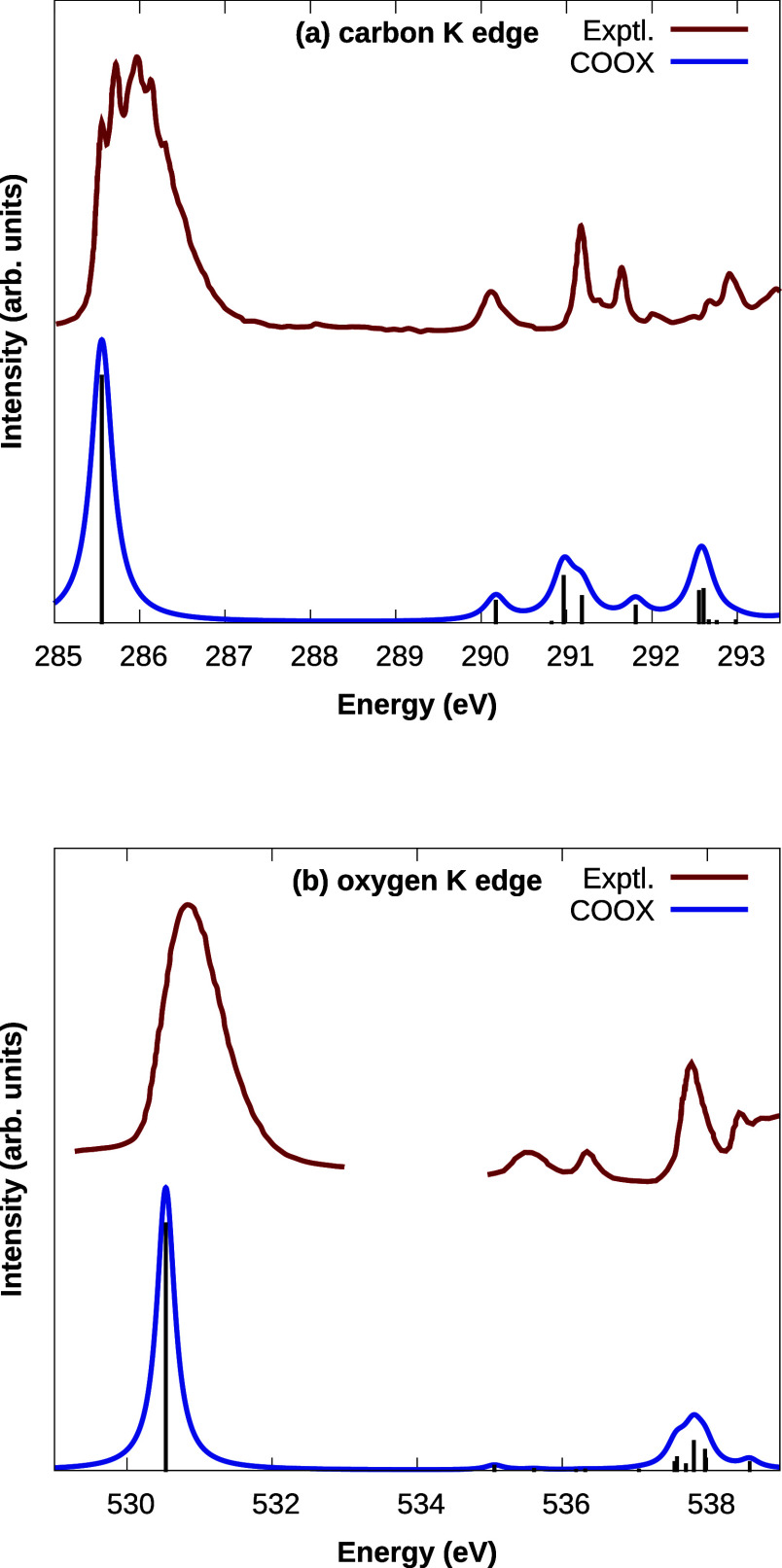
X-ray absorption spectra of formaldehyde near the (a) carbon and (b) oxygen K-edges, computed using COOX with the PBE0 functional and the aug-pcX-2 basis set from 50 TDA roots. Lorentzian broadening with a half width at half-maximum of 0.15 eV has been applied. Experimental spectra digitized with permission from Remmers et al., *Phys. Rev. A***1992,***46,* 3935. Copyright 1992 American Physical Society.

Figure 5 shows X-ray absorption spectra of formaldehyde near the carbon and oxygen K-edges computed using COOX and the PBE0 functional, compared to experimental spectra.^92^ We used the same computational setup as discussed above, only that in this case, we optimized the first 50 TDA roots for each core orbital. While in both cases, the energies of the main peaks are slightly underestimated and some of the more detailed features of the experimental spectra are not reproduced, the overall agreement with the experimental spectra is of similar quality as that of (shifted) CVS coupled-cluster with singles and doubles (CVS-CCSD),^28^ which is especially encouraging considering the low computational expense of COOX compared to higher-level methods like coupled-cluster and the lack of any manual shift of the COOX spectra.

As a final application example for K-edge excitations, we optimized geometries of formic acid for core-excited states from the carbon and (carbonyl) oxygen 1s orbitals with the ωB97X functional using either the Broyden–Fletcher–Goldfarb–Shanno implementation of the ASE package^112^ or the Sella optimizer.^113^ As described in ref (66), we use an unperturbed Kohn–Sham matrix in the computation of the energy-weighted density matrix to form the Pulay correction term. Note that because the optimized density no longer describes a stationary point of the unperturbed Hamiltonian for spin-polarized COOX, this represents an approximation: the perturbed Kohn–Sham matrix and emerging derivative of the constraint potential (see, e.g., eq 28 of ref (63)) are substituted by the unperturbed Kohn–Sham matrix. The ramifications of this approximation are yet to be studied in greater detail, though such an investigation would exceed the scope of this work. The optimized geometric parameters are summarized in Table 1, where we list results from core–valence-separated extended algebraic diagrammatic construction theory to second order (CVS-ADC(2)-x)^29^ for comparison. The results obtained with COOX show overall good agreement with the CVS-ADC(2)-x values, particularly for bond lengths, where the structure of the carbon K-edge excitation is matched nearly perfectly; however, COOX predicts a substantially larger displacement of the carbonyl oxygen atom from the molecular plane in the oxygen K-edge excited state with a dihedral angle of 40.2° as opposed to 11.0° predicted by CVS-ADC(2)-x. Deviations for TDA are generally larger, especially for the oxygen K-edge excitation, where the C–O bond length to the hydroxy group is overestimated substantially, whereas the C=O bond length of the carbonyl group is considerably shorter than what is obtained from CVS-ADC(2)-x. While a more in-depth assessment of the quality of COOX geometries is needed to make definitive statements, these preliminary results are promising for future applications within excited-state dynamics, particularly given the fact that gradients for COOX can be obtained from the regular ground-state routines and do not require solution of Z-vector equations for the computation of wave function response terms.

**Table 1:** Geometry Parameters of Ground and Core-Excited State Geometries of Formic Acid Optimized Using the ωB97X Functional and the aug-pcX-2 Basis Set at the TDA and COOX Levels of Theory, in Comparison to CVS-ADC(2)-x Data from Reference (29)

	ground state		C K-edge			O (carbonyl) K-edge		
	ωB97X	MP2^a^	TDA	COOX	ADC(2)^b^	TDA	COOX	ADC(2)^b^
Bond Lengths (Å)								
C–H	1.10	1.09	1.02	1.03	1.02	1.16	1.08	1.09
C–O (carbonyl)	1.19	1.20	1.20	1.24	1.25	1.26	1.34	1.38
C–O (hydroxy)	1.34	1.34	1.33	1.38	1.38	1.47	1.34	1.32
O–H	0.97	0.97	0.96	0.96	0.97	0.97	0.96	0.97
Angles (deg)								
O–C–O	124.9	125.2	122.8	121.4	121.7	122.7	113.9	111.7
Dihedrals (deg)								
O–C–O–H	0.0	0.0	0.0	0.0	0.0	26.7	40.2	11.0

a cc-pVTZ basis set, data from ref (29).

b CVS-ADC(2)-x, 6-311++G** basis set, data from ref (29).

### Dependence on the Exchange–Correlation Functional

So far, we have observed a tentative correlation between an increasing amount of EXX in the underlying xc functional and more positive mean errors, i.e., higher excitation energies on average. This matches our observation for the description of charge-transfer states with COOX from ref (66), albeit to a far lesser extent. To further quantify these trends, we list selected K-edge excitation energies for different functionals from the first four rungs of Jacob’s ladder, with increasing amounts of EXX for functionals on the fourth rung, in Table 2. At the extreme ends of the EXX scale, we notice a systematic underestimation of excitation energies for the SVWN functional^114,115^ residing on the first rung of Jacob’s ladder (local density approximations) and a systematic overestimation of excitation energies by Hartree–Fock. The generalized gradient approximation PBE functional^106^ ranges from an underestimation of 0.7 eV for the carbon K-edge in CH_4_ to an overestimation of 1.0 eV for the fluorine K-edge in hydrogen fluoride. The meta-GGA functional B97M-V^116^ consistently overshoots the experimental energies, an observation that is shared by Hait and Head-Gordon for ROKS core excitation energies.^59^ Among the hybrid functionals, we indeed find a loose trend toward higher excitation energies with increasing amounts of EXX with the range-separated ωB97X functional^109^ yielding the highest excitation energies of the hybrids in all but one case. An exception to this trend is PBE0,^107,108^ whose excitation energies appear closer to those of the semilocal PBE than those of other hybrids. Noteworthily, PBE and PBE0 yield the best agreement with the experimental data, making them good candidates for the cost-effective evaluation of core-excited states in larger systems.

**Table 2:** K-Edge^a^ Excitation Energies (in eV) Computed with COOX and the aug-pcX-2 Basis Set (Including Scalar-Relativistic ZORA Correction)

functional	% EXX	CH_4_	NH_3_	H_2_O	HF	HCOF		
						C	O	F
LDA								
SVWN^114,115^		284.4	397.0	530.2	683.7	285.4	528.0	683.0
GGA								
PBE^106^		287.3	400.6	534.3	688.4	288.2	531.9	687.6
mGGA								
B97M-V^116^		290.0	403.3	537.2	691.5	290.7	534.4	690.4
Global Hybrid								
B3LYP^117^	20	288.2	401.2	534.7	688.6	288.8	532.2	688.1
PBE0^107,108^	25	287.6	400.6	534.1	687.8	288.1	531.6	687.5
PW6B95^118^	28	288.5	401.5	535.1	688.9	289.1	532.6	688.6
BHandHLYP^119^	50	288.4	401.3	534.7	688.5	288.9	532.3	688.4
Range-Separated Hybrid								
ωB97X^109^	≤100^b^	288.7	401.7	535.3	689.1	289.0	532.6	688.5
Wave Function-Based								
Hartree–Fock	100	292.0	401.3	534.9	688.5	289.1	532.2	692.4
exptl.		288.0	400.8	534.0	687.4	288.2	532.1	687.7

a First symmetry-allowed transition from 1s orbitals.

b 15.771% short-range EXX, 100% long-range EXX.

### L_2,3_-Edge Excitations of Third-Period Atoms

We next demonstrate the accuracy of COOX for the 2p excitations of third-period atoms in small molecules from ref (59) shown in Figure 2b, referenced against experimental data from refs (120–136) (see Tables S5–S10 in the Supporting Information for individual references). For these excitations, spin–orbit coupling breaks the energetic degeneracy of the three p-orbitals, giving rise to two distinct energy levels with total angular momentum *j* = 3/2 and *j* = 1/2, respectively. Excitations from these 2p_3/2_ and 2p_1/2_ orbitals are referred to as L_3_ and L_2_, and are measured experimentally with an area ratio of 2:1. We account for this effect in a semiempirical fashion as described in the Supporting Information.

**Figure 6:**
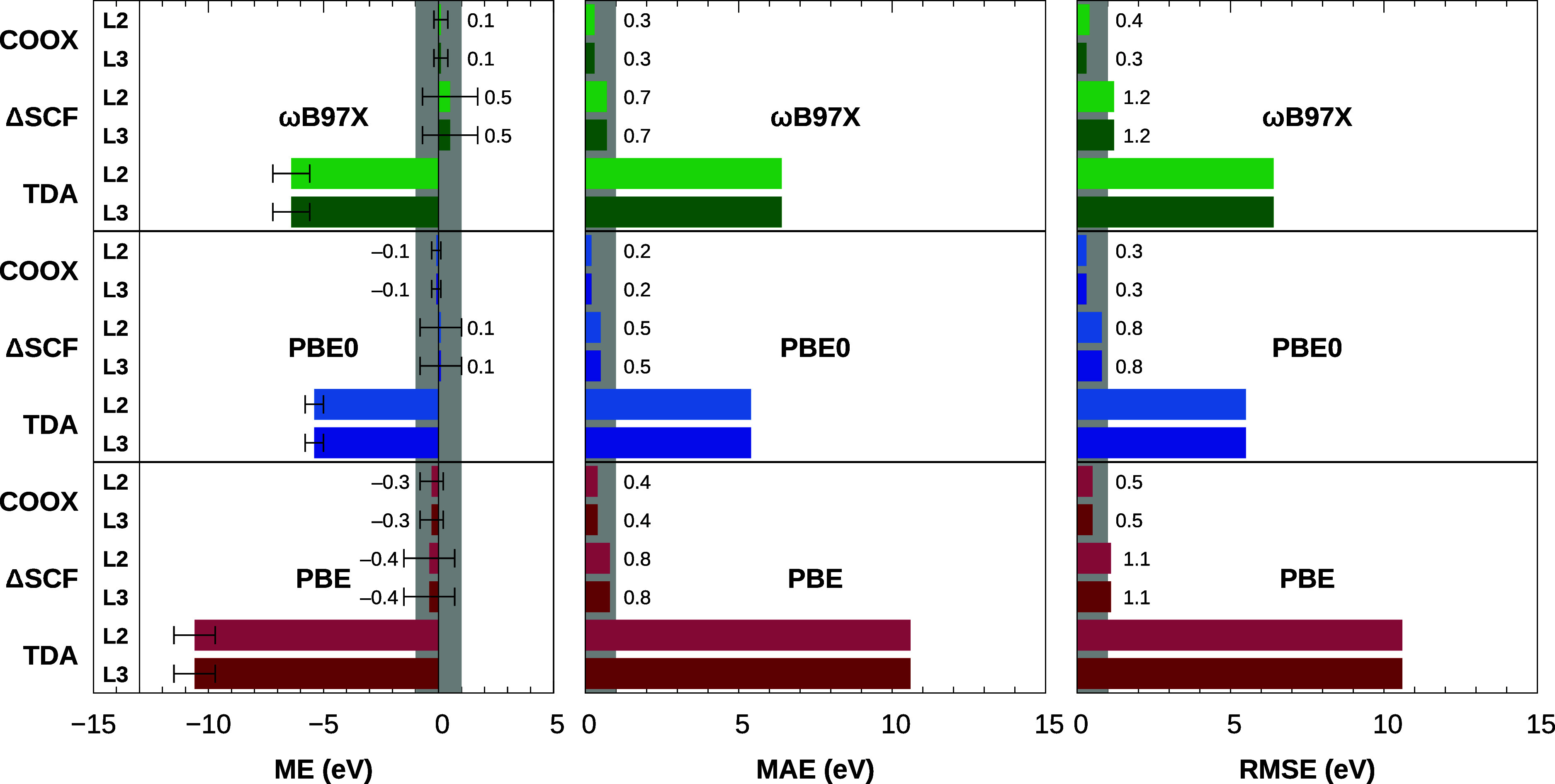
Mean errors with standard deviation (error bars), mean absolute errors, and root mean squared errors (in eV) for L_2,3_-edge excitation energies of third-period elements in small molecules computed at the TDA, ΔSCF, and COOX levels of theory with the PBE, PBE0 and ωB97X functionals using the aug-pcX-2 basis set including scalar-relativistic ZORA corrections and a semiempirical treatment of spin–orbit coupling (see Computational Details for more information). An error region of ±1 eV is shaded in gray.

Figure 6 shows mean errors, mean absolute errors, and root mean squared errors for the 40 L_2_ and L_3_ excitations analyzed in ref (59) computed using TDA, ΔSCF, and COOX with the PBE, PBE0, and ωB97X functionals, including a scalar-relativistic ZORA correction (see Tables S6 and S9 of the Supporting Information for individual excitation energies, Tables S5 and S8 for results without scalar-relativistic corrections, and Tables S7 and S10 for results using scaled-ZORA). Once again, we observe the characteristic underestimation of the excitation energy by TDA, with respective mean errors for the L_3_- and L_2_-edges of −10.6 eV/–10.6 eV for the semilocal PBE functional, −5.4 eV/–5.4 eV for the global hybrid PBE0 functional, and −6.4 eV/–6.4 eV for the range-separated ωB97X functional. The accuracy is considerably improved for the ΔSCF approach, with mean errors reaching as low as 0.1 eV for the PBE0 functional; however, root mean squared errors for the PBE and ωB97X functionals fail to fall under the 1 eV barrier, and mean absolute errors are more than twice as large as those obtained with COOX. The ΔSCF ansatz also shows a far greater variance for L-edge excitations with standard deviations of roughly 1 eV compared to 0.2–0.5 eV for COOX. Furthermore, it should be stressed a number of excited states failed to converge outright using ΔSCF (see Tables S5–S10 of the Supporting Information); these states are thus excluded for the computation of ME, MAE, and RMSE for ΔSCF only. In contrast, COOX not only offers stable convergence for all excited states investigated, but also accounts for the lowest errors in all considered metrics. The best results are obtained with PBE0 with a very low ME of −0.1 eV, MAE of 0.2 eV, and RMSE of 0.3 eV, respectively.

### L- and M-Edge Excitations of Heavier Atoms

To demonstrate the robustness of the COOX ansatz, we computed L_2,3_ and M_4,5_ excitation energies for a series of heavier elements from the fourth to seventh period, comprising molecules containing vanadium, chromium, molybdenum, palladium, tungsten, rhenium, and uranium, with geometries for all molecules taken from ref (137). For these systems, the ZORA Hamiltonian is no longer an appropriate choice for describing scalar-relativistic effects due to large errors in the inner core region compared to the four-component Dirac–Coulomb Hamiltonian;^138^ we therefore report energies computed with the scaled-ZORA variant^90^ instead, which more closely matches the accuracy of more sophisticated relativistic methods like the X2C Hamiltonian.^138^ Energies computed without scalar-relativistic corrections and with the unscaled ZORA variant are once again included in the Supporting Information. Furthermore, it should be noted that within the framework of cDFT, certain modifications must be made for the calculation of the scaled-ZORA energy correction, which we describe within the Supporting Information.

Excitation energies for the L_2,3_ and/or M_4,5_ edges of these systems are shown in Table 3, once again making use of the semiempirical inclusion of spin–orbit coupling. Where available, we use the aug-cc-pwCVTZ-DK basis set^76,77^ (using the non-DK variant for lighter atoms). While this basis set is optimized for the Douglas–Kroll–Hess Hamiltonian, we have verified its suitability for our calculations employing scaled-ZORA by comparing to calculations in which the basis set was fully decontracted. For molecules containing sixth- and seventh-period atoms, we use the ma-ZORA-def2-TZVPP basis set from the ORCA library^78^ in conjunction with the SARC-ZORA-TZVPP basis set^79,80^ for the heavy atoms W, Re, and U, as described in the Computational Details section. Regarding Table 3, the shortcomings of ΔSCF become immediately apparent, as even with use of the maximum overlap method, the overwhelming majority of ΔSCF calculations failed to converge or, in some cases, suffered from variational collapse—in contrast, all COOX calculations converged without issues.

**Table 3:** L_2,3_- and M_4,5_-Edge Excitations of Heavy Atoms in Small Molecules^a^^,^^b^^,^^c^ Computed Using ΔSCF and COOX Compared to Experimental Values (in eV)

				PBE		PBE0			ωB97X	
molecule			exptl.	ΔSCF	COOX	4c^d^	ΔSCF	COOX	ΔSCF	COOX
VOCl_3_	e	L_3_	516.9^141^	g	514.5	507.5	g	516.2	g	516.1
		L_2_	523.8^141^	g	521.4	514.2	g	523.0	g	523.0
CrO_2_Cl_2_	e	L_3_	579.9^141^	576.7	577.0	571.4	578.7	578.0	578.4	578.6
		L_2_	588.5^141^	585.3	585.6	579.5	587.3	586.6	587.0	587.2
MoS_4_^2–^	e	L_3_	2521.7^142^	g	2532.5	2489.3	g	2536.4	g	2535.5
		L_2_	2620.6^142^	g	2631.4	2595.9	g	2635.3	g	2634.4
		M_5_	228.7^142^	h	231.4	225.4	232.1	231.9	g	232.0
		M_4_	231.7^142^	h	234.4	228.7	235.1	234.9	g	235.0
PdCl_6_^2–^	e	L_3_	3177.8^143^	h	3187.1	3138.2	h	3191.6	h	3190.1
		L_2_	3334.7^143^	h	3344.0	3297.4	h	3348.5	h	3347.0
WCl_6_	f	L_3_	10,212.2^144^	g	10,272.9	10,139.8	g	10,283.7	g	10,280.7
		L_2_	11,547.1^144^	g	11,607.8	11,492.8	g	11,618.6	g	11,615.6
ReO_4_^–^	f	L_3_	10,542.0^145^	g	10,600.8	10,472.0	g	10,611.6	g	10,608.4
		L_2_		g	12,041.9	11,911.9	g	12,052.7	g	12,049.5
UO_2_(NO_3_)_2_	f	M_5_		g	3622.5	3515.3	3642.2	3630.6	3640.1	3628.4
		M_4_	3727.0^146^	g	3800.6	3693.2	3820.3	3808.7	3818.2	3806.5

a Lowest symmetry-allowed transition from 2p and 3d orbitals, respectively.

b Including scalar-relativistic scaled-ZORA correction.

c Including semiempirical treatment of spin–orbit coupling (see Supporting Information).

d Data from ref (137), decontracted Dyall-VDZ/aug-cc-pVDZ basis set.

e aug-cc-pwCVTZ-DK basis set.

f ma-ZORA-def2-TZVPP/SARC-ZORA-TZVPP basis set.

g Excited-state calculation did not converge.

h Variational collapse.

In terms of accuracy, we observe larger errors than previously obtained; in particular, for the L-edge excitations of WCl_6_ and ReO_4_^–^, errors compared to experiment reach up to ∼70 eV, corresponding to about 0.6% of the total excitation energy, whereas the M_5_-edge excitation of uranyl nitrate is overestimated by as much as 81.3 eV for PBE0 (2.2% of the total excitation energy). Deviations between the investigated Kohn–Sham functionals are comparatively small, especially between the hybrid PBE0 and ωB97X functionals, likely pointing toward a systematic issue rather than inaccuracies in the individual functionals. The excitation energies of above 10 keV range well into the hard X-ray regime, in which the validity of the dipole approximation becomes highly questionable, and other contributions such as the electric quadrupole moment are expected to start playing an important role.^9,10^ While this will only impact the predicted intensities of the excitations, missing relativistic effects may impact the accuracy of the respective excitation energies substantially, as for the heavier atoms, the magnitude of these effects is much greater in terms of spin–orbit splittings (cf. Table S4 in the Supporting Information) and scalar-relativistic contributions. Thus, a more sophisticated description of relativistic effects may be required to improve accuracy, such as the spin-free exact two-component Hamiltonian (SFX2C)^139^ or potentially other variants of X2C which also include a description of spin–orbit interaction.^138,140^ Comparison between COOX and ΔSCF (for the cases where ΔSCF does converge) again show only small deviations between the two methods, suggesting that both methods converge to identical or at least similar energetic saddle points. Finally, Table 3 also includes results from fully relativistic four-component (4c) calculations with the PBE0 functional and basis sets of double-ζ quality (decontracted Dyall-VDZ and aug-cc-pVDZ) from ref (137). We note in passing that these basis sets are likely too far from the complete basis set limit to make meaningful comparisons to experimental data; in fact, we observe a systematic underestimation of the excitation energies which we would expect to become less severe if larger basis sets were employed. Furthermore, the vastly differently sized basis sets used for 4c and COOX mean we cannot undertake a direct comparison of the results. However, qualitatively, COOX excitations seem to provide satisfactory results when referenced against the much more involved 4c ansatz, albeit a systematic overestimation of excitation energies is observed, which again may indicate the need for a more accurate description of relativistic effects.

## Conclusion and Outlook

We have adapted our constraint-based orbital-optimized excited-state method for the computation of core excitation energies, introducing a spin-polarized version of COOX that allows us to optimize core excitations with a single constraint for both the mixed-spin excitation and the triplet excitation used for spin purification, mimicking the general procedure adopted in ΔSCF calculations.

We have benchmarked our method on a set of experimental K-, L-, and M-edge excitation energies of small systems, and have shown COOX to deliver highly accurate results for K- and L-edge excitations of second- and third-period atoms with average errors well below 1 eV when combined with scalar-relativistic ZORA and a semiempirical treatment of spin–orbit coupling. The overall accuracy achieved with COOX is comparable to that of the popular ΔSCF method without requiring a rather involved manual assignment of orbital transitions. COOX was further shown to be able to produce X-ray absorption spectra that show good qualitative and quantitative agreement with experimental spectra without the need to apply any manual energy shift despite the constraint being based on amplitudes from linear-response theory, for which shifting is a de facto requirement. Excited-state geometry optimizations can be carried out using unaltered routines for analytic ground-state gradients and have been shown to yield structures that are—in contrast to optimized TDA structures—in overall good agreement with higher-level methods such as CVS-ADC(2)-x.

We have shown COOX to offer robust convergence with standard DIIS algorithms and tight convergence thresholds even for core excitations of heavier atoms for which ΔSCF routinely failed to converge or encountered variational collapse. We thus believe COOX to be a highly attractive method for future orbital-optimized studies of core-excited states given its comparatively simple nature, low computational cost, and algorithmic robustness, although some obstacles still remain which warrant further investigation: the necessary spin purification is an obvious drawback, and it would be desirable to be able to use a spin-restricted COOX variant which offers the same accuracy and stability, either in the form of a closed-shell ansatz with Fermi smearing as is being used for local excitations, or following a restricted open-shell ansatz akin to ROKS/SGM. Furthermore, a more rigorous treatment of relativistic effects may be desirable for future applications: on the one hand, we observed accuracies deteriorate for L-edge excitations of sixth- and seventh-period elements, which may indicate a requirement to move beyond ZORA for the description of scalar-relativistic effects, although the scaled-ZORA energy correction was found to still yield satisfactory results due to a more accurate description of the deep core region compared to plain ZORA.^138^ On the other hand, the reliance on experimental data for treating spin–orbit coupling for excitations from orbitals with  > 0 is certainly dissatisfying, and its description within the confinements of COOX—either through the one-electron approximation of Gordon and co-workers^147^ for the estimation of splitting constants or through a fully relativistic treatment of spin–orbit interaction within a two-component scheme—should be investigated to overcome this shortcoming and turn COOX into a true ab initio method for arbitrary core excitations.

## Supporting Information Available

Method for semiempirical spin–orbit coupling; scaled-ZORA energy terms for COOX; individual excitation energies computed in this work (with and without scalar-relativistic ZORA and scaled-ZORA); experimental spin–orbit splittings used for L- and M-edge excitation energies (PDF)
