# Supplementary material for: Highly Accurate and Robust Constraint-Based Orbital-Optimized Core Excitations
Source: J Phys Chem A. 2024 Nov 4;128(45):9804–18. doi: 10.1021/acs.jpca.4c04139 (PMC11571214; doi:10.1021/acs.jpca.4c04139)
Supplement: Supplementary file 1 — jp4c04139_si_001.pdf [file jp4c04139_si_001.pdf]

# Supporting Information: Highly Accurate and Robust Constraint-Based Orbital-Optimized Core Excitations

Yannick Lemke,† Jörg Kussmann,*,† and Christian Ochsenfeld*,†,‡

†*Chair of Theoretical Chemistry, Department of Chemistry, Ludwig-Maximilians-Universität München, Butenandtstr. 5–13, D-81377 Munich, Germany*

‡*Max-Planck-Institute for Solid State Research, Heisenbergstr. 1, D-70569 Stuttgart, Germany*

E-mail: joerg.kussmann@uni-muenchen.de; christian.ochsenfeld@uni-muenchen.de

## Contents

**1 Semi-Empirical Treatment of Spin–Orbit Coupling for $L_{2,3}$- and $M_{4,5}$-Edge Excitations S2**

**2 Scaled-ZORA Energy Correction for cDFT S4**

**3 K-Edge Excitations of Second-Period Atoms S5**

**4 Experimental Spin–Orbit Splittings S8**

**5 L-Edge Excitations of Third-Period Atoms S9**

**6 L- and M-Edge Excitations of Heavier Atoms S15**

**References S17**

# 1 Semi-Empirical Treatment of Spin–Orbit Coupling for $L_{2,3}$- and $M_{4,5}$-Edge Excitations

For excitations from 2p and 3d orbitals, we incorporate the effects of spin–orbit coupling using the semi-empirical method of Hait and Head-Gordon,[1] in which a matrix representation of the spin–orbit coupling operator

$$\hat{H}_{\text{soc}} = -J(\hat{L}_+\hat{S}_- + \hat{L}_-\hat{S}_+ + \hat{L}_z\hat{S}_z) \tag{S1}$$

is constructed and diagonalized. In the basis of p-orbitals ($\ell = 1$), this matrix reads as

$$\mathbf{H}_{\text{soc}}^{\ell=1} = -\mathrm{i}J \begin{pmatrix} \mathbf{0} & -\boldsymbol{\sigma}_z & \boldsymbol{\sigma}_y \\ \boldsymbol{\sigma}_z & \mathbf{0} & -\boldsymbol{\sigma}_x \\ -\boldsymbol{\sigma}_y & \boldsymbol{\sigma}_x & \mathbf{0} \end{pmatrix} \tag{S2}$$

with the $2 \times 2$ Pauli spin matrices $\boldsymbol{\sigma}_{x,y,z}$, from which we extract a submatrix of the interacting $|\mathrm{p}_z\rangle \otimes |\alpha\rangle$ and $|\mathrm{p}_{x,y}\rangle \otimes |\beta\rangle$ orbitals and fill the diagonal with the computed excitation energies $\omega_{1,2,3}$ from all three p-orbitals, yielding

$$\mathbf{H}_{\text{soc}}^{\ell=1} = \begin{pmatrix} \omega_1 & J & -\mathrm{i}J \\ J & \omega_2 & -\mathrm{i}J \\ \mathrm{i}J & \mathrm{i}J & \omega_3 \end{pmatrix}. \tag{S3}$$

As noted by Hait and Head-Gordon,[1] in the case of perfect degeneracy between the p orbitals (and thus, $\omega_1 = \omega_2 = \omega_3 = \omega$), diagonalization of this Hamiltonian would yield a twofold degenerate eigenvalue $\omega - J$ ($L_3$-edge) and a third eigenvalue $\omega + 2J$ ($L_2$-edge) in accordance with the characteristic 2:1 area ratio measured in X-ray absorption spectra. In practice, however, this degeneracy is broken by the presence of the molecular field. In our calculations, we follow the procedure of Hait and Head-Gordon,[1] i.e., the two lowest eigenvalues of $\mathbf{H}_{\text{soc}}^{\ell=1}$ are averaged to yield the $L_3$-edge

excitation energy, and an experimentally determined value of $J$ is used to construct $\mathbf{H}_{\text{soc}}^{\ell=1}$ and compute the $L_2$-edge excitation energy as $\omega_{L_2} = \omega_{L_3} + 3J$. The experimental values used for $3J$ are listed in Table S4. An alternative approach which we mention for the sake of completeness would be to estimate splitting constants using the one-electron spin–orbit coupling operator of ref 2 either from TDA/TDDFT amplitudes or directly from the non-orthogonal COOX wavefunctions, though we have not investigated this in detail.

In the basis of d-orbitals ($\ell = 2$), the spin–orbit coupling matrix reads as

$$\mathbf{H}_{\text{soc}}^{\ell=2} = -\mathrm{i}J \begin{pmatrix} \mathbf{0} & \mathbf{0} & \mathbf{0} & -\sqrt{3}\boldsymbol{\sigma}_y & \sqrt{3}\boldsymbol{\sigma}_x \\ \mathbf{0} & \mathbf{0} & -2\boldsymbol{\sigma}_z & \boldsymbol{\sigma}_y & \boldsymbol{\sigma}_x \\ \mathbf{0} & 2\boldsymbol{\sigma}_z & \mathbf{0} & -\boldsymbol{\sigma}_x & \boldsymbol{\sigma}_y \\ \sqrt{3}\boldsymbol{\sigma}_y & -\boldsymbol{\sigma}_y & \boldsymbol{\sigma}_x & \mathbf{0} & -\boldsymbol{\sigma}_z \\ -\sqrt{3}\boldsymbol{\sigma}_x & -\boldsymbol{\sigma}_x & -\boldsymbol{\sigma}_y & \boldsymbol{\sigma}_z & \mathbf{0} \end{pmatrix}, \tag{S4}$$

from which the submatrix of the interacting $|\mathrm{d}_{z^2,x^2-y^2,xy}\rangle \otimes |\alpha\rangle$ and $|\mathrm{d}_{xz,yz}\rangle \otimes |\beta\rangle$ orbitals is extracted and augmented by the computed excitation energies $\omega_{1,\dots,5}$ to obtain

$$\mathbf{H}_{\text{soc}}^{\ell=2} = \begin{pmatrix} \omega_1 & 0 & 0 & \sqrt{3}J & -\mathrm{i}\sqrt{3}J \\ 0 & \omega_2 & 2\mathrm{i}J & -J & -\mathrm{i}J \\ 0 & -2\mathrm{i}J & \omega_3 & \mathrm{i}J & -J \\ \sqrt{3}J & -J & -\mathrm{i}J & \omega_4 & -\mathrm{i}J \\ \mathrm{i}\sqrt{3}J & \mathrm{i}J & -J & \mathrm{i}J & \omega_5 \end{pmatrix}. \tag{S5}$$

Again, in the special case of degenerate d-orbitals ($\omega_1 = \cdots = \omega_5 = \omega$), the eigenvalues simplify to a threefold degenerate eigenvalue of $\omega - 2J$ ($M_5$-edge) and a twofold degenerate eigenvalue of $\omega + 3J$ ($M_4$-edge) in alignment with the experimentally observed 3:2 area ratio of the $M_5$ and $M_4$ peaks. Like for p-orbitals, we construct $\mathbf{H}_{\text{soc}}^{\ell=2}$ using experimental values of $J$ (values for which are listed in Table S4), average the three lowest eigenvalues to obtain $\omega_{M_5}$, and compute the $M_4$-edge excitation energy as $\omega_{M_4} = \omega_{M_5} + 5J$.

## 2 Scaled-ZORA Energy Correction for cDFT

For the excitation energies listed in Table 3 of the main text, we employed the scalar-relativistic scaled-ZORA Hamiltonian,[3] which gives rise to the same eigenfunctions as the regular scalar-relativistic ZORA Hamiltonian, but leads to different orbital energies and a different total energy of the system. Typically, the scaled orbital energies are obtained using the relation

$$\varepsilon_p^{\text{sc-ZORA}} = \varepsilon_p^{\text{ZORA}} \times \frac{1}{1 + \langle \varphi_p | \hat{\mathbf{p}} \frac{c^2}{(2c^2 - V_{\text{MP}})^2} \hat{\mathbf{p}} | \varphi_p \rangle}, \tag{S6}$$

where $\hat{\mathbf{p}}$ is the momentum operator, $c$ is the speed of light (in atomic units), and $V_{\text{MP}}$ is the model potential of van Wüllen,[4] giving rise to the total energy correction

$$\begin{aligned} E^{\text{sc-ZORA}} &= E^{\text{ZORA}} + \sum_{i=1}^{N} \varepsilon_i^{\text{sc-ZORA}} - \sum_{i=1}^{N} \varepsilon_i^{\text{ZORA}} \\ &= E^{\text{ZORA}} - \sum_{i=1}^{N} \varepsilon_i^{\text{ZORA}} \times \frac{\langle \varphi_i | \hat{\mathbf{p}} \frac{c^2}{(2c^2 - V_{\text{MP}})^2} \hat{\mathbf{p}} | \varphi_i \rangle}{1 + \langle \varphi_i | \hat{\mathbf{p}} \frac{c^2}{(2c^2 - V_{\text{MP}})^2} \hat{\mathbf{p}} | \varphi_i \rangle}. \end{aligned} \tag{S7}$$

In the case of cDFT, however, some special precautions must be taken because the orbital energies $\varepsilon_p^{\text{ZORA}}$ also include the constraint term, i.e.,

$$\varepsilon_p^{\text{ZORA@cDFT}} = \langle \varphi_p | \hat{F}^{\text{KS}} | \varphi_p \rangle = \langle \varphi_p | \hat{F}_0^{\text{KS}} | \varphi_p \rangle + \lambda_{\text{c}} \langle \varphi_p | W_{\text{c}} | \varphi_p \rangle, \tag{S8}$$

where $\hat{F}_0^{\text{KS}}$ is the regular Kohn–Sham Hamiltonian. The energy corrections given in eqs S6 and S7 are therefore not correct for cDFT; instead, we define the following corrections:

$$\varepsilon_p^{\text{sc-ZORA@cDFT}} = \langle \varphi_p | \hat{F}_0^{\text{KS}} | \varphi_p \rangle \times \frac{1}{1 + \langle \varphi_p | \hat{\mathbf{p}} \frac{c^2}{(2c^2 - V_{\text{MP}})^2} \hat{\mathbf{p}} | \varphi_p \rangle} + \lambda_{\text{c}} \langle \varphi_p | W_{\text{c}} | \varphi_p \rangle \tag{S9}$$

$$E^{\text{sc-ZORA@cDFT}} = E^{\text{ZORA@cDFT}} - \sum_{i=1}^{N} \langle \varphi_i | \hat{F}_0^{\text{KS}} | \varphi_i \rangle \times \frac{\langle \varphi_i | \hat{\mathbf{p}} \frac{c^2}{(2c^2 - V_{\text{MP}})^2} \hat{\mathbf{p}} | \varphi_i \rangle}{1 + \langle \varphi_i | \hat{\mathbf{p}} \frac{c^2}{(2c^2 - V_{\text{MP}})^2} \hat{\mathbf{p}} | \varphi_i \rangle}. \tag{S10}$$

# 3 K-Edge Excitations of Second-Period Atoms

Table S1: K-edge excitation energies of second-row atoms[a] computed at TDA, COOX,[b] and ΔSCF[b] levels of theory (without relativistic corrections) with the aug-pcX-2 basis set (in eV).

| | | | PBE | | | PBE0 | | | ωB97X | | |
|---|---|---|---|---|---|---|---|---|---|---|---|
| molecule | ref. | exptl. | TDA | COOX | ΔSCF | TDA | COOX | ΔSCF | TDA | COOX | ΔSCF |
| $\mathbf{C}_2H_4$ | 5 | 284.7 | 268.1 | 284.3 | 283.8 | 275.2 | 284.1 | 284.1 | 273.4 | 285.1 | 284.9 |
| H**C**HO | 6 | 285.6 | 269.3 | 285.5 | 284.8 | 275.9 | 285.3 | 285.1 | 274.1 | 286.2 | 285.9 |
| $\mathbf{C}_2H_2$ | 5 | 285.9 | 269.0 | 285.2 | 284.7 | 276.2 | 285.1 | 285.0 | 274.4 | 286.1 | 285.8 |
| $\mathbf{C}_2N_2$ | 7 | 286.3 | 268.5 | 285.7 | 285.1 | 276.1 | 285.7 | 285.5 | 274.3 | 286.6 | 286.3 |
| H**C**N | 7 | 286.4 | 269.6 | 286.0 | 285.3 | 276.5 | 285.8 | 285.7 | 274.6 | 286.8 | 286.4 |
| $(CH_3)_2\mathbf{C}O$ | 8 | 286.4 | 270.1 | 286.3 | 285.5 | 276.7 | 286.1 | 285.9 | 274.9 | 286.9 | 286.5 |
| $\mathbf{C}_2H_6$ | 5 | 286.9 | 268.2 | 286.3 | 285.8 | 276.6 | 286.4 | 286.3 | 275.5 | 287.5 | 287.4 |
| **C**O | 9 | 287.4 | 270.5 | 287.2 | 286.1 | 276.9 | 286.9 | 286.5 | 275.0 | 287.8 | 287.1 |
| $\mathbf{C}H_4$ | 10 | 288.0 | 269.0 | 287.0 | 286.7 | 277.3 | 287.3 | 287.2 | 276.3 | 288.4 | 288.4 |
| $\mathbf{C}H_3OH$ | 8 | 288.0 | 269.4 | 287.5 | 287.0 | 277.8 | 287.7 | 287.6 | 276.6 | 288.9 | 288.7 |
| H**C**OOH | 8 | 288.1 | 271.5 | 287.8 | 287.0 | 278.1 | 287.6 | 287.4 | 276.3 | 288.5 | 288.1 |
| H**C**OF | 11 | 288.2 | 271.7 | 288.0 | 287.1 | 278.3 | 287.8 | 287.6 | 276.5 | 288.7 | 288.3 |
| $\mathbf{C}O_2$ | 12 | 290.8 | 273.8 | 290.3 | 289.1 | 280.4 | 290.0 | 289.8 | 278.7 | 290.9 | 290.4 |
| $\mathbf{C}F_2O$ | 11 | 290.9 | 274.5 | 290.4 | 289.5 | 280.9 | 290.3 | 290.0 | 279.1 | 291.2 | 290.7 |
| $C_2\mathbf{N}_2$ | 7 | 398.9 | 379.1 | 398.3 | 397.7 | 388.0 | 397.9 | 398.0 | 386.0 | 398.9 | 398.8 |
| HC**N** | 7 | 399.7 | 380.5 | 399.1 | 398.5 | 388.8 | 398.8 | 398.8 | 386.6 | 399.8 | 399.6 |
| imidazole (**N**) | 13 | 399.9 | 379.3 | 399.7 | 399.9 | 388.7 | 399.0 | 399.0 | 386.5 | 399.8 | 399.7 |
| $\mathbf{N}H_3$ | 10 | 400.8 | 380.5 | 401.4 | 400.8 | 389.0 | 400.1 | 399.8 | 387.1 | 401.2 | 401.0 |
| $\mathbf{N}_2$ | 14 | 400.9 | 381.7 | 400.3 | 399.7 | 389.7 | 400.1 | 400.0 | 387.4 | 401.1 | 400.7 |
| $N_2O$ (**N**NO) | 15 | 401.0 | 381.7 | 400.3 | 399.8 | 390.1 | 400.1 | 400.0 | 387.8 | 401.0 | 400.8 |
| glycine (**N**) | 16 | 401.2 | 379.6 | 401.3 | 401.2 | 389.7 | 400.8 | 401.7 | 387.9 | 401.9 | 401.7 |
| pyrrole (**N**) | 17 | 402.3 | 381.3 | 401.6 | 401.2 | 391.1 | 401.7 | 401.7 | 388.6 | 402.3 | 402.2 |
| imidazole (**N**H) | 13 | 402.3 | 381.6 | 402.0 | 401.5 | 390.9 | 401.4 | 401.5 | 388.7 | 402.3 | 402.3 |
| $N_2O$ (N**N**O) | 15 | 404.6 | 385.3 | 403.8 | 403.2 | 393.4 | 403.7 | 403.6 | 391.2 | 404.7 | 404.4 |
| HCH**O** | 6 | 530.8 | 508.6 | 530.0 | 529.6 | 518.2 | 529.6 | 529.7 | 515.5 | 530.7 | 530.5 |
| $(CH_3)_2C\mathbf{O}$ | 8 | 531.4 | 508.6 | 530.3 | 529.9 | 518.4 | 529.9 | 530.0 | 515.8 | 531.0 | 530.8 |
| HC**O**F | 11 | 532.1 | 509.4 | 531.0 | 530.6 | 519.1 | 530.7 | 530.7 | 516.4 | 531.7 | 531.6 |
| HC**O**OH | 8 | 532.2 | 509.1 | 530.9 | 530.5 | 519.0 | 530.5 | 530.6 | 516.3 | 531.6 | 531.5 |
| $CF_2\mathbf{O}$ | 11 | 532.7 | 510.2 | 532.0 | 531.6 | 520.0 | 531.6 | 531.7 | 517.3 | 532.7 | 532.6 |
| $H_2\mathbf{O}$ | 10 | 534.0 | 509.6 | 533.4 | 532.5 | 520.6 | 533.2 | 532.9 | 518.0 | 534.4 | 534.1 |
| $CH_3\mathbf{O}H$ | 8 | 534.1 | 509.7 | 533.6 | 532.7 | 520.9 | 533.3 | 533.1 | 518.4 | 534.4 | 534.2 |
| C**O** | 9 | 534.2 | 511.5 | 533.1 | 532.7 | 521.4 | 532.8 | 532.8 | 518.6 | 533.8 | 533.7 |
| $N_2\mathbf{O}$ | 15 | 534.6 | 511.7 | 533.8 | 533.6 | 522.2 | 533.4 | 533.5 | 519.5 | 534.3 | 534.3 |
| furan (**O**) | 18 | 535.2 | 511.5 | 534.0 | 533.7 | 522.0 | 533.8 | 533.9 | 519.3 | 534.8 | 534.8 |
| HCO**O**H | 8 | 535.4 | 510.8 | 533.9 | 533.8 | 521.8 | 534.0 | 534.1 | 519.2 | 535.0 | 535.0 |
| $C\mathbf{O}_2$ | 15 | 535.4 | 511.7 | 535.0 | 534.3 | 522.6 | 534.1 | 534.1 | 519.9 | 535.1 | 535.0 |
| $\mathbf{F}_2$ | 19 | 682.2 | 658.1 | 681.8 | 681.1 | 668.2 | 681.0 | 681.0 | 665.2 | 682.1 | 681.8 |
| H**F** | 19 | 687.4 | 659.2 | 686.9 | 685.7 | 671.6 | 686.4 | 686.0 | 668.4 | 687.6 | 687.1 |
| HCO**F** | 11 | 687.7 | 659.2 | 686.1 | 686.0 | 671.8 | 686.0 | 686.1 | 668.6 | 687.0 | 687.1 |
| $C\mathbf{F}_2O$ | 11 | 689.2 | 660.5 | 687.7 | 688.2 | 673.1 | 687.6 | 687.7 | 670.0 | 688.6 | 688.7 |
| ME | | | −20.8 | −0.6 | −1.2 | −11.8 | −0.9 | −0.9 | −14.0 | 0.1 | −0.1 |
| Std. dev. | | | 3.5 | 0.4 | 0.4 | 1.8 | 0.4 | 0.4 | 2.3 | 0.4 | 0.3 |
| MAE | | | 20.8 | 0.7 | 1.2 | 11.8 | 0.9 | 1.0 | 14.0 | 0.3 | 0.3 |
| RMSE | | | 21.1 | 0.8 | 1.2 | 11.9 | 1.0 | 1.0 | 14.2 | 0.4 | 0.3 |

[a]First symmetry-allowed transition from 1s orbitals of atoms highlighted in bold. [b]Purified using AP.

Table S2: K-edge excitation energies of second-row atoms[a] computed at TDA,[b] COOX,[b,c] and $\Delta$SCF[b,c] levels of theory with the aug-pcX-2 basis set including scalar-relativistic ZORA (in eV).

| | | | PBE | | | PBE0 | | | $\omega$B97X | | |
|---|---|---|---|---|---|---|---|---|---|---|---|
| molecule | ref. | exptl. | TDA | COOX | $\Delta$SCF | TDA | COOX | $\Delta$SCF | TDA | COOX | $\Delta$SCF |
| $\mathbf{C}_2H_4$ | 5 | 284.7 | 268.4 | 284.5 | 284.1 | 275.4 | 284.4 | 284.4 | 273.6 | 285.3 | 285.1 |
| H**C**HO | 6 | 285.6 | 269.5 | 285.7 | 285.1 | 276.2 | 285.6 | 285.4 | 274.3 | 286.5 | 291.1 |
| $\mathbf{C}_2H_2$ | 5 | 285.9 | 269.3 | 285.5 | 284.9 | 276.5 | 285.4 | 285.3 | 274.6 | 286.4 | 286.1 |
| $\mathbf{C}_2N_2$ | 7 | 286.3 | 268.8 | 286.0 | 285.4 | 276.3 | 285.9 | 285.7 | 274.5 | 286.8 | 286.5 |
| H**C**N | 7 | 286.4 | 269.8 | 286.3 | 285.6 | 276.8 | 286.1 | 285.9 | 274.9 | 287.0 | 286.7 |
| $(CH_3)_2\mathbf{C}O$ | 8 | 286.4 | 270.3 | 286.6 | 285.8 | 276.9 | 286.3 | 286.1 | 275.1 | 287.1 | 286.8 |
| $\mathbf{C}_2H_6$ | 5 | 286.9 | 268.5 | 286.6 | 286.0 | 276.8 | 286.7 | 286.5 | 275.7 | 287.8 | 287.7 |
| **C**O | 9 | 287.4 | 270.7 | 287.5 | 286.3 | 277.1 | 287.1 | 286.8 | 275.2 | 288.0 | 287.4 |
| $\mathbf{C}H_4$ | 10 | 288.0 | 269.2 | 287.3 | 287.0 | 277.6 | 287.6 | 287.5 | 276.5 | 288.7 | 288.6 |
| $\mathbf{C}H_3OH$ | 8 | 288.0 | 269.7 | 287.7 | 287.3 | 278.0 | 288.0 | 287.8 | 276.8 | 289.1 | 289.0 |
| H**C**OOH | 8 | 288.1 | 271.7 | 288.1 | 287.2 | 278.3 | 287.9 | 287.7 | 276.5 | 288.8 | 288.3 |
| H**C**OF | 11 | 288.2 | 272.0 | 288.2 | 287.4 | 278.5 | 288.1 | 287.8 | 276.7 | 289.0 | 288.5 |
| $\mathbf{C}O_2$ | 12 | 290.8 | 274.1 | 290.6 | 289.4 | 280.7 | 290.3 | 290.0 | 278.9 | 291.2 | 290.7 |
| $\mathbf{C}F_2O$ | 11 | 290.9 | 274.7 | 290.6 | 289.7 | 281.1 | 290.6 | 290.3 | 279.4 | 291.5 | 291.0 |
| $C_2\mathbf{N}_2$ | 7 | 398.9 | 379.6 | 398.8 | 398.2 | 388.5 | 398.4 | 398.5 | 386.5 | 399.4 | 399.3 |
| HC**N** | 7 | 399.7 | 380.9 | 399.6 | 399.0 | 389.2 | 399.3 | 399.3 | 387.0 | 400.3 | 400.1 |
| imidazole (**N**) | 13 | 399.9 | 379.8 | 400.2 | 400.4 | 389.2 | 399.5 | 399.5 | 387.0 | 400.3 | 400.3 |
| $\mathbf{N}H_3$ | 10 | 400.8 | 380.9 | 401.9 | 401.3 | 389.5 | 400.6 | 400.4 | 387.6 | 401.7 | 401.5 |
| $\mathbf{N}_2$ | 14 | 400.9 | 382.2 | 400.9 | 400.2 | 390.2 | 400.6 | 400.5 | 387.9 | 401.6 | 401.2 |
| $N_2O$ (**N**NO) | 15 | 401.0 | 382.2 | 400.9 | 400.3 | 390.6 | 400.6 | 400.6 | 388.3 | 401.5 | 401.3 |
| glycine (**N**) | 16 | 401.2 | 380.0 | 401.8 | 401.7 | 390.2 | 401.3 | 402.2 | 388.3 | 402.4 | 402.2 |
| pyrrole (**N**) | 17 | 402.3 | 381.7 | 402.1 | 401.7 | 391.6 | 402.2 | 402.2 | 389.7 | 403.6 | 402.7 |
| imidazole (**N**H) | 13 | 402.3 | 382.1 | 402.5 | 402.0 | 391.4 | 401.9 | 402.0 | 389.2 | 402.9 | 402.8 |
| $N_2O$ (N**N**O) | 15 | 404.6 | 385.8 | 404.3 | 403.7 | 393.9 | 404.2 | 404.2 | 391.6 | 405.2 | 404.9 |
| HCH**O** | 6 | 530.8 | 509.4 | 530.9 | 530.5 | 519.0 | 530.5 | 530.6 | 516.3 | 531.6 | 536.3 |
| $(CH_3)_2C\mathbf{O}$ | 8 | 531.4 | 509.4 | 531.2 | 530.8 | 519.3 | 530.8 | 530.9 | 516.6 | 531.9 | 531.7 |
| HC**O**F | 11 | 532.1 | 510.2 | 531.9 | 531.5 | 520.0 | 531.6 | 531.6 | 517.3 | 532.6 | 532.5 |
| HC**O**OH | 8 | 532.2 | 510.0 | 531.8 | 531.4 | 519.9 | 531.4 | 531.5 | 517.1 | 532.5 | 532.4 |
| $CF_2\mathbf{O}$ | 11 | 532.7 | 511.0 | 532.9 | 532.5 | 520.9 | 532.5 | 532.6 | 518.1 | 533.6 | 533.5 |
| $H_2\mathbf{O}$ | 10 | 534.0 | 510.5 | 534.3 | 533.4 | 521.5 | 534.1 | 533.8 | 518.9 | 535.3 | 535.0 |
| $CH_3\mathbf{O}H$ | 8 | 534.1 | 510.5 | 534.5 | 533.6 | 521.8 | 534.2 | 534.0 | 519.2 | 535.3 | 535.1 |
| C**O** | 9 | 534.2 | 512.3 | 534.0 | 533.6 | 522.2 | 533.7 | 533.7 | 519.5 | 534.7 | 534.6 |
| $N_2\mathbf{O}$ | 15 | 534.6 | 512.5 | 534.7 | 534.5 | 523.0 | 534.3 | 534.4 | 520.3 | 535.2 | 535.2 |
| furan (**O**) | 18 | 535.2 | 512.4 | 534.9 | 534.6 | 522.8 | 534.7 | 534.8 | 520.2 | 535.7 | 535.7 |
| HCO**O**H | 8 | 535.4 | 511.6 | 534.8 | 534.7 | 522.6 | 534.9 | 535.0 | 520.0 | 535.9 | 536.0 |
| $C\mathbf{O}_2$ | 15 | 535.4 | 512.6 | 535.9 | 535.2 | 523.5 | 535.0 | 535.0 | 520.8 | 536.0 | 535.9 |
| $\mathbf{F}_2$ | 19 | 682.2 | 659.5 | 683.3 | 682.6 | 669.6 | 682.5 | 682.4 | 666.6 | 683.5 | 683.2 |
| H**F** | 19 | 687.4 | 660.6 | 688.4 | 687.2 | 673.0 | 687.8 | 687.5 | 669.8 | 689.1 | 688.6 |
| HCO**F** | 11 | 687.7 | 660.6 | 687.6 | 687.4 | 673.2 | 687.5 | 687.6 | 670.0 | 688.5 | 688.6 |
| $C\mathbf{F}_2O$ | 11 | 689.2 | 661.9 | 689.2 | 689.6 | 674.6 | 689.1 | 689.2 | 671.4 | 690.0 | 690.2 |
| ME | | | −20.2 | 0.02 | −0.5 | −11.2 | −0.3 | −0.3 | −13.4 | 0.7 | 0.8 |
| Std. dev. | | | 3.2 | 0.4 | 0.5 | 1.4 | 0.2 | 0.3 | 1.9 | 0.3 | 1.1 |
| MAE | | | 20.2 | 0.3 | 0.6 | 11.2 | 0.3 | 0.4 | 13.4 | 0.7 | 0.8 |
| RMSE | | | 20.4 | 0.4 | 0.7 | 11.3 | 0.4 | 0.4 | 13.5 | 0.8 | 1.4 |

[a]First symmetry-allowed transition from 1s orbitals of atoms highlighted in bold. [b]Including scalar-relativistic ZORA correction. [c]Purified using AP.

Table S3: K-edge excitation energies of second-row atoms[a] computed at TDA,[b] COOX,[b,c] and ΔSCF[b,c] levels of theory with the aug-pcX-2 basis set including scalar-relativistic scaled-ZORA (in eV).

| | | | PBE | | | PBE0 | | | ωB97X | | |
|---|---|---|---|---|---|---|---|---|---|---|---|
| molecule | ref. | exptl. | TDA | COOX | ΔSCF | TDA | COOX | ΔSCF | TDA | COOX | ΔSCF |
| **C**$_2$H$_4$ | 5 | 284.7 | 268.3 | 284.4 | 284.0 | 275.3 | 284.3 | 284.3 | 273.5 | 285.2 | 285.1 |
| H**C**HO | 6 | 285.6 | 269.4 | 285.7 | 285.0 | 276.0 | 285.5 | 285.4 | 274.2 | 286.4 | 291.0 |
| **C**$_2$H$_2$ | 5 | 285.9 | 269.2 | 285.4 | 284.9 | 276.3 | 285.3 | 285.2 | 274.5 | 286.3 | 286.0 |
| **C**$_2$N$_2$ | 7 | 286.3 | 268.7 | 285.9 | 285.3 | 276.2 | 285.8 | 285.7 | 274.4 | 286.7 | 286.5 |
| H**C**N | 7 | 286.4 | 269.7 | 286.2 | 285.5 | 276.7 | 286.0 | 285.9 | 274.8 | 287.0 | 286.6 |
| (CH$_3$)$_2$**C**O | 8 | 286.4 | 270.2 | 286.5 | 285.7 | 276.8 | 286.2 | 286.1 | 275.0 | 287.1 | 286.7 |
| **C**$_2$H$_6$ | 5 | 286.9 | 268.3 | 286.5 | 286.0 | 276.7 | 286.6 | 286.5 | 275.6 | 287.7 | 287.6 |
| **C**O | 9 | 287.4 | 270.6 | 287.4 | 286.3 | 277.0 | 287.1 | 286.7 | 275.1 | 288.0 | 287.4 |
| **C**H$_4$ | 10 | 288.0 | 269.1 | 287.2 | 286.9 | 277.5 | 287.5 | 287.4 | 276.4 | 288.6 | 288.6 |
| **C**H$_3$OH | 8 | 288.0 | 269.6 | 287.7 | 287.2 | 277.9 | 287.9 | 287.8 | 276.7 | 289.0 | 289.0 |
| H**C**OOH | 8 | 288.1 | 271.6 | 288.0 | 287.2 | 278.2 | 287.8 | 287.6 | 276.4 | 288.7 | 288.3 |
| H**C**OF | 11 | 288.2 | 271.9 | 288.2 | 287.3 | 278.4 | 288.0 | 287.8 | 276.6 | 288.9 | 288.5 |
| **C**O$_2$ | 12 | 290.8 | 273.9 | 290.5 | 289.3 | 280.6 | 290.2 | 290.0 | 278.8 | 291.1 | 290.6 |
| **C**F$_2$O | 11 | 290.9 | 274.6 | 290.6 | 289.7 | 281.0 | 290.5 | 290.2 | 279.3 | 291.4 | 290.9 |
| C$_2$**N**$_2$ | 7 | 398.9 | 379.4 | 398.7 | 398.1 | 388.3 | 398.3 | 398.4 | 386.3 | 399.3 | 399.2 |
| HC**N** | 7 | 399.7 | 380.7 | 399.5 | 398.9 | 389.0 | 399.2 | 399.2 | 386.8 | 400.2 | 400.0 |
| imidazole (**N**) | 13 | 399.9 | 379.6 | 400.1 | 400.3 | 389.0 | 399.3 | 399.4 | 386.8 | 400.2 | 400.2 |
| **N**H$_3$ | 10 | 400.8 | 380.7 | 401.8 | 401.2 | 389.3 | 400.4 | 400.2 | 387.4 | 401.6 | 401.4 |
| **N**$_2$ | 14 | 400.9 | 381.9 | 400.7 | 400.1 | 389.9 | 400.4 | 400.3 | 387.6 | 401.4 | 401.1 |
| N$_2$O (**N**NO) | 15 | 401.0 | 382.0 | 400.7 | 400.2 | 390.3 | 400.4 | 400.4 | 388.0 | 401.4 | 401.1 |
| glycine (**N**) | 16 | 401.2 | 379.8 | 401.7 | 401.6 | 389.9 | 401.2 | 402.1 | 388.1 | 402.2 | 402.1 |
| pyrrole (**N**) | 17 | 402.3 | 381.5 | 402.0 | 401.6 | 391.3 | 402.0 | 402.1 | 389.5 | 403.4 | 402.6 |
| imidazole (**N**H) | 13 | 402.3 | 381.9 | 402.3 | 402.0 | 391.2 | 401.8 | 401.9 | 389.0 | 402.7 | 402.7 |
| N$_2$O (N**N**O) | 15 | 404.6 | 385.5 | 404.2 | 403.6 | 393.6 | 404.1 | 404.0 | 391.4 | 405.0 | 404.8 |
| HCH**O** | 6 | 530.8 | 509.0 | 530.6 | 530.3 | 518.6 | 530.2 | 530.4 | 515.9 | 531.3 | 536.1 |
| (CH$_3$)$_2$C**O** | 8 | 531.4 | 509.0 | 531.0 | 530.6 | 518.9 | 530.5 | 530.7 | 516.2 | 531.5 | 531.6 |
| HC**O**F | 11 | 532.1 | 509.8 | 531.7 | 531.4 | 519.6 | 531.3 | 531.5 | 516.9 | 532.3 | 532.3 |
| HC**O**OH | 8 | 532.2 | 509.6 | 531.5 | 531.3 | 519.5 | 531.1 | 531.3 | 516.7 | 532.2 | 532.2 |
| CF$_2$**O** | 11 | 532.7 | 510.6 | 532.6 | 532.3 | 520.5 | 532.2 | 532.4 | 517.7 | 533.3 | 533.3 |
| H$_2$**O** | 10 | 534.0 | 510.1 | 534.0 | 533.1 | 521.1 | 533.8 | 533.6 | 518.5 | 535.0 | 534.8 |
| CH$_3$**O**H | 8 | 534.1 | 510.1 | 534.2 | 533.4 | 521.3 | 533.9 | 533.8 | 518.8 | 535.0 | 534.9 |
| C**O** | 9 | 534.2 | 511.9 | 533.7 | 533.5 | 521.8 | 533.4 | 533.6 | 519.1 | 534.4 | 534.4 |
| N$_2$**O** | 15 | 534.6 | 512.1 | 534.4 | 534.3 | 522.6 | 534.0 | 534.3 | 519.9 | 534.9 | 535.0 |
| furan (**O**) | 18 | 535.2 | 512.0 | 534.6 | 534.4 | 522.4 | 534.4 | 534.7 | 519.8 | 535.4 | 535.5 |
| HCO**O**H | 8 | 535.4 | 511.2 | 534.5 | 534.6 | 522.2 | 534.6 | 534.8 | 519.6 | 535.6 | 535.8 |
| C**O**$_2$ | 15 | 535.4 | 512.2 | 535.6 | 535.1 | 523.0 | 534.6 | 534.9 | 520.3 | 535.7 | 535.7 |
| **F**$_2$ | 19 | 682.2 | 658.8 | 682.7 | 682.2 | 668.9 | 682.0 | 682.0 | 665.9 | 683.0 | 682.8 |
| H**F** | 19 | 687.4 | 659.9 | 687.9 | 686.8 | 672.3 | 687.3 | 687.1 | 669.2 | 688.6 | 688.2 |
| HCO**F** | 11 | 687.7 | 659.9 | 687.0 | 687.2 | 672.5 | 686.9 | 687.4 | 669.4 | 688.0 | 688.3 |
| C**F**$_2$O | 11 | 689.2 | 661.3 | 688.6 | 689.4 | 673.9 | 688.5 | 689.0 | 670.7 | 689.5 | 689.9 |
| ME | | | −20.5 | −0.2 | −0.6 | −11.5 | −0.5 | −0.4 | −13.7 | 0.5 | 0.6 |
| Std. dev. | | | 3.3 | 0.4 | 0.4 | 1.6 | 0.2 | 0.3 | 2.1 | 0.3 | 1.1 |
| MAE | | | 20.5 | 0.3 | 0.7 | 11.5 | 0.5 | 0.5 | 13.7 | 0.5 | 0.6 |
| RMSE | | | 20.7 | 0.4 | 0.8 | 11.6 | 0.5 | 0.5 | 13.8 | 0.6 | 1.3 |

[a]First symmetry-allowed transition from 1s orbitals of atoms highlighted in bold. [b]Including scalar-relativistic scaled-ZORA correction (see Section 2). [c]Purified using AP.

# 4 Experimental Spin–Orbit Splittings

Table S4: Experimental values for spin–orbit splittings used for the construction of $\mathbf{H}_{\mathrm{soc}}$ in eV (see Section 1).

| atom | ref. | $L_2$–$L_3$ (3$J$) | $M_4$–$M_5$ (5$J$) |
|---|---|---|---|
| Si | 20 | 0.60 | — |
| P | 20 | 0.80 | — |
| S | 20 | 1.13 | — |
| Cl | 20 | 1.60 | — |
| V | 21 | 6.9 | — |
| Cr | 21 | 8.6 | — |
| Mo | 22 | 98.9 | 3.0 |
| Pd | 23 | 156.9 | — |
| W | 24 | 1334.9 | — |
| Re[a] | 25 | 1441.9 | — |
| U[a] | 25 | — | 178.1 |

[a]Computed 4c@PBE0-xHF (60% exact exchange) splittings from Ref. 25 in lieu of experimental data.

# 5 L-Edge Excitations of Third-Period Atoms

Table S5: $L_3$-edge excitation energies of third-row atoms[a] computed at TDA,[b] COOX,[b,c] and ΔSCF[b,c] levels of theory (without scalar-relativistic corrections) with the aug-pcX-2 basis set (in eV).

| | | | PBE | | | PBE0 | | | ωB97X | | |
|---|---|---|---|---|---|---|---|---|---|---|---|
| molecule | ref. | exptl. | TDA | COOX | ΔSCF | TDA | COOX | ΔSCF | TDA | COOX | ΔSCF |
| **Si**$H_4$ | 26 | 102.6 | 93.4 | 102.8 | 102.2 | 97.9 | 102.9 | 102.7 | 97.3 | 103.0 | 102.9 |
| **Si**$(CH_3)_4$ | 27 | 102.9 | 92.2 | 101.8 | 101.8 | 97.2 | 102.9 | 102.3 | 97.5 | 103.8 | —[d] |
| **Si**$Cl_4$ | 27 | 104.3 | 95.7 | 103.7 | 103.2 | 99.8 | 104.0 | 103.9 | 99.4 | 104.5 | 104.4 |
| **Si**$(OCH_3)_4$ | 28 | 104.8 | 94.1 | 104.1 | —[d] | 99.1 | 104.6 | 104.5 | 99.2 | 105.2 | 105.1 |
| **Si**$F_4$ | 29 | 106.1 | 96.0 | 105.6 | 104.9 | 100.5 | 106.0 | 105.6 | 100.1 | 106.3 | 106.0 |
| **P**$H_3$ | 30 | 131.9 | 122.0 | 132.3 | 131.8 | 126.7 | 131.8 | 131.7 | 125.6 | 132.1 | 131.9 |
| **P**$(CH_3)_3$ | 30 | 132.3 | 121.6 | 132.1 | 132.0 | 127.0 | 132.2 | —[d] | 126.1 | 132.5 | —[d] |
| **P**$F_3$ | 31 | 134.9 | 125.2 | 134.9 | 134.2 | 129.5 | 135.0 | 134.7 | 128.4 | 135.2 | 134.9 |
| O**P**$F_3$ | 31 | 137.1 | 126.3 | 136.6 | 135.7 | 131.4 | 137.0 | 136.7 | 130.6 | 137.2 | 136.9 |
| **P**$F_5$ | 32 | 138.2 | 127.7 | 137.9 | 136.8 | 132.7 | 138.1 | 137.7 | 132.0 | 138.3 | 138.0 |
| C**S**$_2$ | 33 | 163.3 | 152.6 | 162.7 | 162.4 | 157.8 | 162.7 | 165.0 | 156.6 | 162.6 | 164.9 |
| (**S**$CH_3)_2$ | 34 | 164.1 | 153.6 | 163.6 | —[d] | 158.5 | 163.7 | —[d] | 157.4 | 163.9 | 166.7 |
| OC**S** | 35 | 164.3 | 153.6 | 163.8 | 163.6 | 158.8 | 163.8 | 163.8 | 157.5 | 163.8 | 163.7 |
| $H_2$**S** | 36 | 164.4 | 153.8 | 164.9 | 164.2 | 159.2 | 164.7 | 164.3 | 158.0 | 164.8 | 164.8 |
| **S**$F_6$ | 37 | 172.3 | 161.8 | 171.1 | 170.6 | 166.7 | 171.8 | 171.7 | 165.8 | 172.0 | 171.9 |
| **Cl**$_2$ | 38 | 198.2 | 187.3 | 198.0 | 197.9 | 192.3 | 197.9 | 200.7 | 190.9 | 197.9 | 201.2 |
| C**Cl**$_4$ | 39 | 200.3 | 188.0 | 200.4 | 203.8 | 194.0 | 199.9 | 201.9 | 192.6 | 200.0 | 203.2 |
| H**Cl** | 40 | 200.6 | 189.0 | 200.8 | 200.0 | 194.7 | 200.7 | 200.5 | 193.4 | 200.8 | 200.7 |
| $C_6H_5$**Cl** | 41 | 201.5 | 188.4 | 201.5 | 201.4 | 195.1 | 200.9 | 201.6 | 193.7 | 201.2 | 201.3 |
| **Cl**$F_3$ | 42 | 201.8 | 191.0 | 201.3 | 200.9 | 196.0 | 201.5 | 201.4 | 194.6 | 201.7 | 201.5 |
| ME | | | −10.6 | −0.3 | −0.5 | −5.5 | −0.2 | 0.1 | −6.5 | 0.1 | 0.5 |
| Std. dev. | | | 1.0 | 0.5 | 1.1 | 0.5 | 0.3 | 0.9 | 0.8 | 0.4 | 1.2 |
| MAE | | | 10.6 | 0.4 | 0.8 | 5.5 | 0.3 | 0.5 | 6.5 | 0.3 | 0.7 |
| RMSE | | | 10.7 | 0.6 | 1.1 | 5.6 | 0.3 | 0.8 | 6.5 | 0.4 | 1.2 |

[a]First symmetry-allowed transition from 2p orbitals of atoms highlighted in bold. [b]Including semi-empirical treatment of spin–orbit coupling (see Section 1). [c]Purified using AP. [d]Excited-state calculation did not converge.

Table S6: $L_3$-edge excitation energies of third-row atoms[a] computed at TDA,[b,c] COOX,[b–d] and ΔSCF[b–d] levels of theory with the aug-pcX-2 basis set including scalar-relativistic ZORA (in eV).

| molecule | ref. | exptl. | PBE TDA | PBE COOX | PBE ΔSCF | PBE0 TDA | PBE0 COOX | PBE0 ΔSCF | ωB97X TDA | ωB97X COOX | ωB97X ΔSCF |
|---|---|---|---|---|---|---|---|---|---|---|---|
| **Si**$H_4$ | 26 | 102.6 | 93.4 | 102.8 | 102.2 | 97.9 | 102.9 | 102.7 | 97.3 | 103.0 | 103.0 |
| **Si**$(CH_3)_4$ | 27 | 102.9 | 92.2 | 101.8 | 101.8 | 97.9 | 103.0 | 102.3 | 98.3 | 103.8 | —[e] |
| **Si**$Cl_4$ | 27 | 104.3 | 95.6 | 103.6 | 103.2 | 99.7 | 103.9 | 103.9 | 99.4 | 104.4 | 104.3 |
| **Si**$(OCH_3)_4$ | 28 | 104.8 | 94.1 | 104.1 | 104.5 | 99.1 | 104.6 | 104.5 | 99.2 | 105.2 | 105.1 |
| **Si**$F_4$ | 29 | 106.1 | 96.0 | 105.6 | 104.8 | 100.5 | 105.9 | 105.6 | 100.1 | 106.3 | 106.0 |
| **P**$H_3$ | 30 | 131.9 | 122.0 | 132.3 | 131.8 | 126.8 | 131.9 | 131.8 | 125.6 | 132.1 | 132.0 |
| **P**$(CH_3)_3$ | 30 | 132.3 | 121.7 | 132.1 | 132.1 | 127.1 | 132.2 | —[e] | 126.1 | 132.5 | —[e] |
| **P**$F_3$ | 31 | 134.9 | 125.2 | 134.9 | 134.2 | 129.5 | 135.0 | 134.7 | 128.5 | 135.2 | 134.9 |
| O**P**$F_3$ | 31 | 137.1 | 126.3 | 136.6 | 135.7 | 131.4 | 137.0 | 136.7 | 130.6 | 137.2 | 136.9 |
| **P**$F_5$ | 32 | 138.2 | 127.7 | 137.9 | 136.7 | 132.7 | 138.0 | 137.7 | 132.0 | 138.2 | 137.9 |
| C**S**$_2$ | 33 | 163.3 | 152.7 | 162.8 | 162.6 | 157.9 | 162.8 | 165.1 | 156.7 | 162.7 | 165.0 |
| (**S**$CH_3)_2$ | 34 | 164.1 | 153.6 | 163.7 | —[e] | 158.6 | 163.8 | —[e] | 157.4 | 164.0 | 166.7 |
| OC**S** | 35 | 164.3 | 153.7 | 163.9 | 163.7 | 158.9 | 163.9 | 163.9 | 157.6 | 163.9 | 163.8 |
| $H_2$**S** | 36 | 164.4 | 153.9 | 165.0 | 164.3 | 159.3 | 164.8 | 164.4 | 158.1 | 164.9 | 164.9 |
| **S**$F_6$ | 37 | 172.3 | 161.7 | 171.0 | 170.6 | 166.7 | 171.7 | 171.7 | 165.7 | 172.0 | —[e] |
| **Cl**$_2$ | 38 | 198.2 | 187.4 | 198.2 | 198.0 | 192.4 | 198.1 | 200.9 | 191.1 | 198.1 | 201.3 |
| C**Cl**$_4$ | 39 | 200.3 | 188.1 | 200.5 | 203.9 | 194.1 | 200.0 | 201.6 | 192.7 | 200.1 | 203.4 |
| H**Cl** | 40 | 200.6 | 189.1 | 200.9 | 200.2 | 194.9 | 200.8 | 200.7 | 193.5 | 201.0 | 200.8 |
| $C_6H_5$**Cl** | 41 | 201.5 | 188.5 | 201.6 | 201.6 | 195.2 | 201.6 | 201.8 | 193.9 | 201.4 | 201.5 |
| **Cl**$F_3$ | 42 | 201.8 | 191.1 | 201.4 | 201.1 | 196.1 | 201.7 | 201.6 | 194.7 | 201.8 | 201.6 |
| ME | | | −10.6 | −0.3 | −0.4 | −5.4 | −0.1 | 0.1 | −6.4 | 0.1 | 0.5 |
| Std. dev. | | | 0.9 | 0.5 | 1.1 | 0.4 | 0.2 | 0.9 | 0.8 | 0.3 | 1.2 |
| MAE | | | 10.6 | 0.4 | 0.8 | 5.4 | 0.2 | 0.5 | 6.4 | 0.3 | 0.7 |
| RMSE | | | 10.6 | 0.5 | 1.1 | 5.5 | 0.3 | 0.8 | 6.4 | 0.3 | 1.2 |

[a]First symmetry-allowed transition from 2p orbitals of atoms highlighted in bold. [b]Including semi-empirical treatment of spin–orbit coupling (see Section 1). [c]Including scalar-relativistic ZORA correction. [d]Purified using AP. [e]Excited-state calculation did not converge.

Table S7: $L_3$-edge excitation energies of third-row atoms[a] computed at TDA,[b,c] COOX,[b–d] and ΔSCF[b–d] levels of theory with the aug-pcX-2 basis set including scalar-relativistic scaled-ZORA (in eV).

| | | | PBE | | | PBE0 | | | ωB97X | | |
|---|---|---|---|---|---|---|---|---|---|---|---|
| molecule | ref. | exptl. | TDA | COOX | ΔSCF | TDA | COOX | ΔSCF | TDA | COOX | ΔSCF |
| **Si**$H_4$ | 26 | 102.6 | 93.4 | 103.0 | 102.5 | 97.9 | 103.1 | 102.9 | 97.2 | 103.2 | 103.1 |
| **Si**$(CH_3)_4$ | 27 | 102.9 | 92.2 | 102.0 | 102.0 | 97.9 | 103.2 | 102.5 | 98.3 | 104.0 | —[e] |
| **Si**$Cl_4$ | 27 | 104.3 | 95.6 | 103.9 | 103.4 | 99.7 | 104.1 | 104.1 | 99.3 | 104.6 | 104.5 |
| **Si**$(OCH_3)_4$ | 28 | 104.8 | 94.1 | 104.3 | 104.8 | 99.1 | 104.8 | 104.7 | 99.2 | 105.4 | 105.3 |
| **Si**$F_4$ | 29 | 106.1 | 96.0 | 105.8 | 105.1 | 100.5 | 106.2 | 105.8 | 100.0 | 106.5 | 106.2 |
| **P**$H_3$ | 30 | 131.9 | 122.0 | 132.6 | 132.1 | 126.7 | 132.1 | 132.0 | 125.6 | 132.4 | 132.2 |
| **P**$(CH_3)_3$ | 30 | 132.3 | 121.6 | 132.4 | 132.4 | 127.0 | 132.5 | —[e] | 126.0 | 132.8 | —[e] |
| **P**$F_3$ | 31 | 134.9 | 125.1 | 135.2 | 134.5 | 129.5 | 135.3 | 135.0 | 128.4 | 135.5 | 135.2 |
| O**P**$F_3$ | 31 | 137.1 | 126.3 | 136.9 | 136.0 | 131.4 | 137.2 | 136.9 | 130.6 | 137.5 | 137.2 |
| **P**$F_5$ | 32 | 138.2 | 127.7 | 138.2 | 137.0 | 132.7 | 138.3 | 137.9 | 131.9 | 138.5 | 138.2 |
| C**S**$_2$ | 33 | 163.3 | 152.7 | 163.1 | 162.9 | 157.9 | 163.1 | 165.4 | 156.6 | 163.0 | 165.3 |
| (**S**$CH_3)_2$ | 34 | 164.1 | 153.6 | 164.0 | —[e] | 158.5 | 164.1 | —[e] | 157.4 | 164.2 | 167.0 |
| OC**S** | 35 | 164.3 | 153.6 | 164.2 | 163.9 | 158.8 | 164.2 | 164.2 | 157.5 | 164.2 | 164.1 |
| $H_2$**S** | 36 | 164.4 | 153.8 | 165.3 | 164.6 | 159.2 | 165.1 | 164.7 | 158.0 | 165.2 | 165.2 |
| **S**$F_6$ | 37 | 172.3 | 161.6 | 171.3 | 170.9 | 166.6 | 172.0 | 172.0 | 165.6 | 172.3 | —[e] |
| **Cl**$_2$ | 38 | 198.2 | 187.3 | 198.5 | 198.4 | 192.3 | 198.4 | 201.2 | 191.0 | 198.4 | 201.7 |
| C**Cl**$_4$ | 39 | 200.3 | 188.0 | 200.9 | 204.3 | 194.0 | 200.4 | 201.9 | 192.6 | 200.5 | 203.8 |
| H**Cl** | 40 | 200.6 | 189.0 | 201.3 | 200.6 | 194.8 | 201.2 | 201.1 | 193.4 | 201.4 | 201.2 |
| $C_6H_5$**Cl** | 41 | 201.5 | 188.4 | 202.0 | 202.0 | 195.1 | 202.0 | 202.2 | 193.8 | 201.8 | 201.9 |
| **Cl**$F_3$ | 42 | 201.8 | 191.0 | 201.8 | 201.4 | 196.0 | 202.0 | 201.9 | 194.6 | 202.2 | 202.0 |
| ME | | | −5.4 | 0.0 | −0.2 | −2.8 | 0.2 | 0.2 | −6.4 | 0.2 | 0.7 |
| Std. dev. | | | 0.9 | 0.5 | 1.1 | 0.4 | 0.3 | 0.8 | 0.8 | 0.3 | 1.2 |
| MAE | | | 5.4 | 0.4 | 0.7 | 2.8 | 0.2 | 0.3 | 6.4 | 0.2 | 0.8 |
| RMSE | | | 7.6 | 0.5 | 1.1 | 3.9 | 0.3 | 0.8 | 6.5 | 0.4 | 1.4 |

[a]First symmetry-allowed transition from 2p orbitals of atoms highlighted in bold. [b]Including semi-empirical treatment of spin–orbit coupling (see Section 1). [c]Including scalar-relativistic scaled-ZORA correction (see Section 2). [d]Purified using AP. [e]Excited-state calculation did not converge.

Table S8: $L_2$-edge excitation energies of third-row atoms[a] computed at TDA,[b] COOX,[b,c] and ΔSCF[b,c] levels of theory (without scalar-relativistic corrections) with the aug-pcX-2 basis set (in eV).

| | | | PBE | | | PBE0 | | | ωB97X | | |
|---|---|---|---|---|---|---|---|---|---|---|---|
| molecule | ref. | exptl. | TDA | COOX | ΔSCF | TDA | COOX | ΔSCF | TDA | COOX | ΔSCF |
| **Si**$H_4$ | 26 | 103.2 | 94.0 | 103.4 | 102.8 | 98.5 | 103.5 | 103.3 | 97.9 | 103.6 | 103.5 |
| **Si**$(CH_3)_4$ | 27 | 103.5 | 92.8 | 102.4 | 102.4 | 97.8 | 103.5 | 102.9 | 98.1 | 104.4 | —[d] |
| **Si**$Cl_4$ | 27 | 104.9 | 96.3 | 104.3 | 103.8 | 100.4 | 104.6 | 104.5 | 100.0 | 105.1 | 105.0 |
| **Si**$(OCH_3)_4$ | 28 | 105.4 | 94.7 | 104.7 | —[d] | 99.7 | 105.2 | 105.1 | 99.8 | 105.8 | 105.7 |
| **Si**$F_4$ | 29 | 106.7 | 96.6 | 106.2 | 105.5 | 101.1 | 106.6 | 106.2 | 100.7 | 106.9 | 106.6 |
| **P**$H_3$ | 30 | 132.8 | 122.8 | 133.1 | 132.6 | 127.5 | 132.6 | 132.5 | 126.4 | 132.9 | 132.7 |
| **P**$(CH_3)_3$ | 30 | 133.1 | 122.4 | 132.9 | 132.8 | 127.8 | 133.0 | —[d] | 126.9 | 133.3 | —[d] |
| **P**$F_3$ | 31 | 135.6 | 126.0 | 135.7 | 135.0 | 130.3 | 135.8 | 135.5 | 129.2 | 136.0 | 135.7 |
| O**P**$F_3$ | 31 | 137.8 | 127.1 | 137.4 | 136.5 | 132.2 | 137.8 | 137.5 | 131.4 | 138.0 | 137.7 |
| **P**$F_5$ | 32 | 139.0 | 128.5 | 138.7 | 137.6 | 133.5 | 138.9 | 138.5 | 132.8 | 139.1 | 138.8 |
| C**S**$_2$ | 33 | 164.4 | 153.8 | 163.9 | 163.6 | 159.0 | 163.9 | 166.2 | 157.8 | 163.8 | 166.1 |
| (**S**$CH_3)_2$ | 34 | 165.4 | 154.8 | 164.8 | —[d] | 159.7 | 164.9 | —[d] | 158.6 | 165.1 | 167.9 |
| OC**S** | 35 | 165.5 | 154.8 | 165.0 | 164.8 | 160.0 | 165.0 | 165.0 | 158.7 | 165.0 | 164.9 |
| $H_2$**S** | 36 | 165.6 | 155.0 | 166.1 | 165.4 | 160.4 | 165.9 | 165.5 | 159.2 | 166.0 | 166.0 |
| **S**$F_6$ | 37 | 173.4 | 163.0 | 172.3 | 171.8 | 167.9 | 173.0 | 172.9 | 167.0 | 173.2 | 173.1 |
| **Cl**$_2$ | 38 | 199.8 | 188.9 | 199.6 | 199.5 | 193.9 | 199.5 | 202.3 | 192.5 | 199.5 | 202.8 |
| C**Cl**$_4$ | 39 | 201.9 | 189.6 | 202.0 | 205.4 | 195.6 | 201.5 | 203.5 | 194.2 | 201.6 | 204.8 |
| H**Cl** | 40 | 202.4 | 190.6 | 202.4 | 201.6 | 196.3 | 202.3 | 202.1 | 195.0 | 202.4 | 202.3 |
| $C_6H_5$**Cl** | 41 | 203.2 | 190.0 | 203.1 | 203.0 | 196.7 | 202.5 | 203.2 | 195.3 | 202.8 | 202.9 |
| **Cl**$F_3$ | 42 | 203.2 | 192.6 | 202.9 | 202.5 | 197.6 | 203.1 | 203.0 | 196.2 | 203.3 | 203.1 |
| ME | | | −10.6 | −0.3 | −0.5 | −5.5 | −0.2 | 0.1 | −6.4 | 0.1 | 0.5 |
| Std. dev. | | | 1.0 | 0.4 | 1.1 | 0.5 | 0.3 | 0.9 | 0.8 | 0.4 | 1.1 |
| MAE | | | 10.6 | 0.4 | 0.8 | 5.5 | 0.3 | 0.5 | 6.4 | 0.3 | 0.7 |
| RMSE | | | 10.7 | 0.5 | 1.1 | 5.5 | 0.3 | 0.8 | 6.5 | 0.4 | 1.2 |

[a]First symmetry-allowed transition from 2p orbitals of atoms highlighted in bold. [b]Including semi-empirical treatment of spin–orbit coupling (see Section 1). [c]Purified using AP. [d]Excited-state calculation did not converge.

Table S9: $L_2$-edge excitation energies of third-row atoms[a] computed at TDA,[b,c] COOX,[b–d] and ΔSCF[b–d] levels of theory with the aug-pcX-2 basis set including scalar-relativistic ZORA (in eV).

| | | | PBE | | | PBE0 | | | ωB97X | | |
|---|---|---|---|---|---|---|---|---|---|---|---|
| molecule | ref. | exptl. | TDA | COOX | ΔSCF | TDA | COOX | ΔSCF | TDA | COOX | ΔSCF |
| **Si**$H_4$ | 26 | 103.2 | 94.0 | 103.4 | 102.8 | 98.5 | 103.5 | 103.3 | 97.9 | 103.6 | 103.6 |
| **Si**$(CH_3)_4$ | 27 | 103.5 | 92.8 | 102.4 | 102.4 | 98.5 | 103.6 | 102.9 | 98.9 | 104.4 | —[e] |
| **Si**$Cl_4$ | 27 | 104.9 | 96.2 | 104.2 | 103.8 | 100.3 | 104.5 | 104.5 | 100.0 | 105.0 | 104.9 |
| **Si**$(OCH_3)_4$ | 28 | 105.4 | 94.7 | 104.7 | 105.1 | 99.7 | 105.2 | 105.1 | 99.8 | 105.8 | 105.7 |
| **Si**$F_4$ | 29 | 106.7 | 96.6 | 106.2 | 105.4 | 101.1 | 106.5 | 106.2 | 100.7 | 106.9 | 106.6 |
| **P**$H_3$ | 30 | 132.8 | 122.8 | 133.1 | 132.6 | 127.6 | 132.7 | 132.6 | 126.4 | 132.9 | 132.8 |
| **P**$F_3$ | 30 | 135.6 | 122.5 | 135.7 | 135.0 | 127.9 | 135.8 | 135.5 | 126.9 | 136.0 | 135.7 |
| **P**$(CH_3)_3$ | 31 | 133.1 | 126.0 | 132.9 | 132.9 | 130.3 | 133.0 | —[e] | 129.3 | 133.3 | —[e] |
| O**P**$F_3$ | 31 | 137.8 | 127.1 | 137.4 | 136.5 | 132.2 | 137.8 | 137.5 | 131.4 | 138.0 | 137.7 |
| **P**$F_5$ | 32 | 139.0 | 128.5 | 138.7 | 137.5 | 133.5 | 138.8 | 138.5 | 132.8 | 139.0 | 138.7 |
| C**S**$_2$ | 33 | 164.4 | 153.9 | 164.0 | 163.8 | 159.1 | 164.0 | 166.3 | 157.9 | 163.9 | 166.2 |
| (**S**$CH_3)_2$ | 34 | 165.4 | 154.8 | 164.9 | —[e] | 159.8 | 165.0 | —[e] | 158.6 | 165.2 | 167.9 |
| OC**S** | 35 | 165.5 | 154.9 | 165.1 | 164.9 | 160.1 | 165.1 | 165.1 | 158.8 | 165.1 | 165.0 |
| $H_2$**S** | 36 | 165.6 | 155.1 | 166.2 | 165.5 | 160.5 | 166.0 | 165.6 | 159.3 | 166.1 | 166.1 |
| **S**$F_6$ | 37 | 173.4 | 162.9 | 172.2 | 171.8 | 167.9 | 172.9 | 172.9 | 166.9 | 173.2 | —[e] |
| **Cl**$_2$ | 38 | 199.8 | 189.0 | 199.8 | 199.6 | 194.0 | 199.7 | 202.5 | 192.7 | 199.7 | 202.9 |
| C**Cl**$_4$ | 39 | 201.9 | 189.7 | 202.1 | 205.5 | 195.7 | 201.6 | 203.2 | 194.3 | 201.7 | 205.0 |
| H**Cl** | 40 | 202.4 | 190.7 | 202.5 | 201.8 | 196.5 | 202.4 | 202.3 | 195.1 | 202.6 | 202.4 |
| $C_6H_5$**Cl** | 41 | 203.2 | 190.1 | 203.2 | 203.2 | 196.8 | 203.2 | 203.4 | 195.5 | 203.0 | 203.1 |
| **Cl**$F_3$ | 42 | 203.2 | 192.7 | 203.0 | 202.7 | 197.7 | 203.3 | 203.2 | 196.3 | 203.4 | 203.2 |
| ME | | | −10.6 | −0.3 | −0.4 | −5.4 | −0.1 | 0.1 | −6.4 | 0.1 | 0.5 |
| Std. dev. | | | 0.9 | 0.5 | 1.1 | 0.4 | 0.2 | 0.9 | 0.8 | 0.3 | 1.2 |
| MAE | | | 10.6 | 0.4 | 0.8 | 5.4 | 0.2 | 0.5 | 6.4 | 0.3 | 0.7 |
| RMSE | | | 10.6 | 0.5 | 1.1 | 5.5 | 0.3 | 0.8 | 6.4 | 0.4 | 1.2 |

[a]First symmetry-allowed transition from 2p orbitals of atoms highlighted in bold. [b]Including semi-empirical treatment of spin–orbit coupling (see Section 1). [c]Including scalar-relativistic ZORA correction. [d]Purified using AP. [e]Excited-state calculation did not converge.

Table S10: $L_2$-edge excitation energies of third-row atoms[a] computed at TDA,[b,c] COOX,[b–d] and ΔSCF[b–d] levels of theory with the aug-pcX-2 basis set including scalar-relativistic scaled-ZORA (in eV).

| | | | PBE | | | PBE0 | | | ωB97X | | |
|---|---|---|---|---|---|---|---|---|---|---|---|
| molecule | ref. | exptl. | TDA | COOX | ΔSCF | TDA | COOX | ΔSCF | TDA | COOX | ΔSCF |
| **Si**$H_4$ | 26 | 103.2 | 94.0 | 103.6 | 103.1 | 98.5 | 103.7 | 103.5 | 97.8 | 103.8 | 103.7 |
| **Si**$(CH_3)_4$ | 27 | 103.5 | 92.8 | 102.6 | 102.6 | 98.5 | 103.8 | 103.1 | 98.9 | 104.6 | —[e] |
| **Si**$Cl_4$ | 27 | 104.9 | 96.2 | 104.5 | 104.0 | 100.3 | 104.7 | 104.7 | 99.9 | 105.2 | 105.1 |
| **Si**$(OCH_3)_4$ | 28 | 105.4 | 94.7 | 104.9 | 105.4 | 99.7 | 105.4 | 105.3 | 99.8 | 106.0 | 105.9 |
| **Si**$F_4$ | 29 | 106.7 | 96.6 | 106.4 | 105.7 | 101.1 | 106.8 | 106.4 | 100.6 | 107.1 | 106.8 |
| **P**$H_3$ | 30 | 132.8 | 122.8 | 133.4 | 132.9 | 127.5 | 132.9 | 132.8 | 126.4 | 133.2 | 133.0 |
| **P**$F_3$ | 30 | 135.6 | 122.4 | 133.2 | 133.2 | 127.8 | 133.3 | —[e] | 126.8 | 133.6 | —[e] |
| **P**$(CH_3)_3$ | 31 | 133.1 | 125.9 | 136.0 | 135.3 | 130.3 | 136.1 | 135.8 | 129.2 | 136.3 | 136.0 |
| O**P**$F_3$ | 31 | 137.8 | 127.1 | 137.7 | 136.8 | 132.2 | 138.0 | 137.7 | 131.4 | 138.3 | 138.0 |
| **P**$F_5$ | 32 | 139.0 | 128.5 | 139.0 | 137.8 | 133.5 | 139.1 | 138.7 | 132.7 | 139.3 | 139.0 |
| C**S**$_2$ | 33 | 164.4 | 153.9 | 164.3 | 164.1 | 159.1 | 164.3 | 166.6 | 157.8 | 164.2 | 166.5 |
| (**S**$CH_3)_2$ | 34 | 165.4 | 154.8 | 165.2 | —[e] | 159.7 | 165.3 | —[e] | 158.6 | 165.4 | 168.2 |
| OC**S** | 35 | 165.5 | 154.8 | 165.4 | 165.1 | 160.0 | 165.4 | 165.4 | 158.7 | 165.4 | 165.3 |
| $H_2$**S** | 36 | 165.6 | 155.0 | 166.5 | 165.8 | 160.4 | 166.3 | 165.9 | 159.2 | 166.4 | 166.4 |
| **S**$F_6$ | 37 | 173.4 | 162.8 | 172.5 | 172.1 | 167.8 | 173.2 | 173.2 | 166.8 | 173.5 | —[e] |
| **Cl**$_2$ | 38 | 199.8 | 188.9 | 200.1 | 200.0 | 193.9 | 200.0 | 202.8 | 192.6 | 200.0 | 203.3 |
| C**Cl**$_4$ | 39 | 201.9 | 189.6 | 202.5 | 205.9 | 195.6 | 202.0 | 203.5 | 194.2 | 202.1 | 205.4 |
| H**Cl** | 40 | 202.4 | 190.6 | 202.9 | 202.2 | 196.4 | 202.8 | 202.7 | 195.0 | 203.0 | 202.8 |
| $C_6H_5$**Cl** | 41 | 203.2 | 190.0 | 203.6 | 203.6 | 196.7 | 203.6 | 203.8 | 195.4 | 203.4 | 203.5 |
| **Cl**$F_3$ | 42 | 203.2 | 192.6 | 203.4 | 203.0 | 197.6 | 203.6 | 203.5 | 196.2 | 203.8 | 203.6 |
| ME | | | −5.4 | 0.03 | −0.2 | −5.5 | 0.2 | 0.2 | −6.4 | 0.4 | 0.7 |
| Std. dev. | | | 1.0 | 0.5 | 1.1 | 0.5 | 0.2 | 0.8 | 0.8 | 0.3 | 1.2 |
| MAE | | | 5.4 | 0.4 | 0.6 | 5.5 | 0.2 | 0.3 | 6.4 | 0.4 | 0.8 |
| RMSE | | | 7.6 | 0.5 | 1.1 | 5.5 | 0.3 | 0.8 | 6.5 | 0.5 | 1.4 |

[a]First symmetry-allowed transition from 2p orbitals of atoms highlighted in bold. [b]Including semi-empirical treatment of spin–orbit coupling (see Section 1). [c]Including scalar-relativistic scaled-ZORA correction (see Section 2). [d]Purified using AP. [e]Excited-state calculation did not converge.

# 6 L- and M-Edge Excitations of Heavier Atoms

Table S11: $L_{2,3}$- and $M_{4,5}$-edge excitations of heavy atoms in small molecules[a,b] computed using ΔSCF and COOX (without scalar-relativistic corrections) compared to experimental values (in eV).

| molecule | | ref. | | exptl. | PBE ΔSCF | PBE COOX | PBE0 ΔSCF | PBE0 COOX | ωB97X ΔSCF | ωB97X COOX |
|---|---|---|---|---|---|---|---|---|---|---|
| $VOCl_3$ | c | 21 | $L_3$ | 516.9 | —[e] | 511.2 | 512.7 | 512.8 | —[e] | 512.8 |
| | | | $L_2$ | 523.8 | —[e] | 518.1 | 519.6 | 519.7 | —[e] | 519.7 |
| $CrO_2Cl_2$ | c | 21 | $L_3$ | 579.9 | 572.7 | 573.2 | 574.6 | 575.0 | 574.3 | 574.8 |
| | | | $L_2$ | 588.8 | 581.3 | 581.8 | 583.2 | 583.6 | 582.9 | 583.4 |
| $MoS_4^{2-}$ | c | 22 | $L_3$ | 2521.7 | —[e] | 2477.3 | —[e] | 2481.6 | —[e] | 2480.6 |
| | | | $L_2$ | 2620.6 | —[e] | 2576.2 | —[e] | 2580.5 | —[e] | 2579.5 |
| | | | $M_5$ | 228.7 | —[e] | 233.0 | 233.8 | 233.7 | —[e] | 233.8 |
| | | | $M_4$ | 231.7 | —[e] | 236.0 | 236.8 | 236.7 | —[e] | 236.8 |
| $PdCl_6^{2-}$ | c | 23 | $L_3$ | 3177.8 | —[e] | 3107.2 | —[e] | 3111.8 | —[e] | 3110.3 |
| | | | $L_2$ | 3334.7 | —[e] | 3264.1 | —[e] | 3268.7 | —[e] | 3267.2 |
| $WCl_6$ | d | 24 | $L_3$ | 10212.2 | —[e] | 9044.7 | —[e] | 9052.6 | —[e] | 9041.1 |
| | | | $L_2$ | 11547.1 | —[e] | 10379.6 | —[e] | 10387.5 | —[e] | 10376.0 |
| $ReO_4^-$ | d | 43 | $L_3$ | 10542.0 | —[e] | 9297.8 | —[e] | 9304.4 | —[e] | 9299.8 |
| | | | $L_2$ | — | —[e] | 10738.9 | —[e] | 10745.5 | —[e] | 10740.9 |
| $UO_2(NO_3)_2$ | d | 44 | $M_5$ | — | 3550.8 | 3523.7 | —[e] | —[e] | —[e] | —[e] |
| | | | $M_4$ | 3727.0 | 3728.9 | 3701.8 | —[e] | —[e] | —[e] | —[e] |

[a]Lowest symmetry-allowed transition from 2p and 3d orbitals, respectively. [b]Including semi-empirical treatment of spin–orbit coupling (see Section 1). [c]aug-cc-pwCVTZ-DK basis set.
[d]ma-ZORA-def2-TZVPP/SARC-ZORA-TZVPP basis set. [e]Excited-state calculation did not converge.

Table S12: $L_{2,3}$- and $M_{4,5}$-edge excitations of heavy atoms in small molecules[a–c] computed using ΔSCF and COOX including scalar-relativistic ZORA compared to experimental values (in eV).

| molecule | | ref. | | exptl. | PBE ΔSCF | PBE COOX | PBE0 ΔSCF | PBE0 COOX | ωB97X ΔSCF | ωB97X COOX |
|---|---|---|---|---|---|---|---|---|---|---|
| $VOCl_3$ | [d] | 21 | $L_3$ | 516.9 | —[f] | 513.8 | —[f] | 515.4 | —[f] | 515.4 |
| | | | $L_2$ | 523.8 | —[f] | 520.7 | —[f] | 522.3 | —[f] | 522.3 |
| $CrO_2Cl_2$ | [d] | 21 | $L_3$ | 579.9 | 575.5 | 576.2 | 577.6 | 578.0 | 577.3 | 577.9 |
| | | | $L_2$ | 588.5 | 584.3 | 584.8 | 586.2 | 586.6 | 585.9 | 586.4 |
| $MoS_4^{2-}$ | [d] | 22 | $L_3$ | 2521.7 | —[f] | 2530.4 | —[f] | 2534.9 | —[f] | 2533.9 |
| | | | $L_2$ | 2620.6 | —[f] | 2629.3 | —[f] | 2633.8 | —[f] | 2632.8 |
| | | | $M_5$ | 228.7 | —[g] | 228.3 | 229.3 | 229.2 | —[f] | 229.3 |
| | | | $M_4$ | 231.7 | —[g] | 231.3 | 232.3 | 232.2 | —[f] | 232.3 |
| $PdCl_6^{2-}$ | [d] | 23 | $L_3$ | 3177.8 | —[f] | 3186.9 | —[f] | 3191.6 | —[g] | 3190.0 |
| | | | $L_2$ | 3334.7 | —[f] | 3343.8 | —[f] | 3348.5 | —[g] | 3346.9 |
| $WCl_6$ | [e] | 24 | $L_3$ | 10212.2 | —[f] | 10335.7 | —[f] | 10347.2 | —[f] | 10343.8 |
| | | | $L_2$ | 11547.1 | —[f] | 11670.6 | —[f] | 11682.1 | —[f] | 11678.7 |
| $ReO_4^-$ | [e] | 43 | $L_3$ | 10542.0 | —[f] | 10668.8 | —[f] | 10680.5 | —[f] | 10676.9 |
| | | | $L_2$ | — | —[f] | 12109.9 | —[f] | 12121.6 | —[f] | 12118.0 |
| $UO_2(NO_3)_2$ | [e] | 44 | $M_5$ | — | —[f] | 3571.6 | 3572.9 | 3605.1 | 3577.7 | 3602.9 |
| | | | $M_4$ | 3727.0 | —[f] | 3749.7 | 3758.0 | 3783.2 | 3755.8 | 3781.0 |

[a]Lowest symmetry-allowed transition from 2p and 3d orbitals, respectively. [b]Including scalar-relativistic ZORA correction. [c]Including semi-empirical treatment of spin–orbit coupling (see Section 1). [d]aug-cc-pwCVTZ-DK basis set. [e]ma-ZORA-def2-TZVPP/SARC-ZORA-TZVPP basis set. [f]Excited-state calculation did not converge. [g]Variational collapse.

# References


(1) Hait, D.; Head-Gordon, M. Highly Accurate Prediction of Core Spectra of Molecules at Density Functional Theory Cost: Attaining Sub-electronvolt Error from a Restricted Open-Shell Kohn–Sham Approach. *J. Phys. Chem. Lett.* **2020**, *11*, 775–786.

(2) Koseki, S.; Schmidt, M. W.; Gordon, M. S. MCSCF/6-31G(d,p) calculations of one-electron spin-orbit coupling constants in diatomic molecules. *J. Phys. Chem.* **1992**, *96*, 10768–10772.

(3) van Lenthe, J.; Faas, S.; Snijders, J. Gradients in the ab initio scalar zeroth-order regular approximation (ZORA) approach. *Chem. Phys. Lett.* **2000**, *328*, 107–112.

(4) van Wüllen, C. Molecular density functional calculations in the regular relativistic approximation: Method, application to coinage metal diatomics, hydrides, fluorides and chlorides, and comparison with first-order relativistic calculations. *J. Chem. Phys.* **1998**, *109*, 392–399.

(5) Hitchcock, A. P.; Brion, C. E. Carbon K-shell excitation of $C_2H_2$, $C_2H_4$, $C_2H_6$ and $C_6H_6$ by 2.5 keV electron impact. *J. Electron Spectrosc. Relat. Phenom.* **1977**, *10*, 317–330.

(6) Remmers, G.; Domke, M.; Puschmann, A.; Mandel, T.; Xue, C.; Kaindl, G.; Hudson, E.; Shirley, D. A. High-resolution K-shell photoabsorption in formaldehyde. *Phys. Rev. A* **1992**, *46*, 3935–3944.

(7) Hitchcock, A. P.; Brion, C. E. Inner shell electron energy loss studies of HCN and $C_2N_2$. *Chem. Phys.* **1979**, *37*, 319–331.

(8) Prince, K. C.; Richter, R.; de Simone, M.; Alagia, M.; Coreno, M. Near Edge X-ray Absorption Spectra of Some Small Polyatomic Molecules. *J. Phys. Chem. A* **2003**, *107*, 1955–1963.

(9) Sodhi, R. N. S.; Brion, C. E. Reference energies for inner shell electron energy-loss spectroscopy. *J. Electron Spectrosc. Relat. Phenom.* **1984**, *34*, 363–372.

(10) Schirmer, J.; Trofimov, A. B.; Randall, K. J.; Feldhaus, J.; Bradshaw, A. M.; Ma, Y.; Chen, C. T.; Sette, F. K-shell excitation of the water, ammonia, and methane molecules using high-resolution photoabsorption spectroscopy. *Phys. Rev. A* **1993**, *47*, 1136–1147.

(11) Robin, M. B.; Ishii, I.; McLaren, R.; Hitchcock, A. P. Fluorination effects on the inner-shell spectra of unsaturated molecules. *J. Electron Spectrosc. Relat. Phenom.* **1988**, *47*, 53–92.

(12) Tronc, M.; King, G. C.; Read, F. H. Carbon K-shell excitation in small molecules by high-resolution electron impact. *J. Phys. B: At. Mol. Phys.* **1979**, *12*, 137.

(13) Apen, E.; Hitchcock, A. P.; Gland, J. L. Experimental studies of the core excitation of imidazole, 4,5-dicyanoimidazole, and *s*-triazine. *J. Phys. Chem.* **1993**, *97*, 6859–6866.

(14) Myhre, R. H.; Wolf, T. J. A.; Cheng, L.; Nandi, S.; Coriani, S.; Gühr, M.; Koch, H. A theoretical and experimental benchmark study of core-excited states in nitrogen. *J. Chem. Phys.* **2018**, *148*, 064106.

(15) Prince, K. C.; Avaldi, L.; Coreno, M.; Camilloni, R.; de Simone, M. Vibrational structure of core to Rydberg state excitations of carbon dioxide and dinitrogen oxide. *J. Phys. B: At., Mol. Opt. Phys.* **1999**, *32*, 2551.

(16) Plekan, O.; Feyer, V.; Richter, R.; Coreno, M.; de Simone, M.; Prince, K. C.; Carravetta, V. An X-ray absorption study of glycine, methionine and proline. *J. Electron Spectrosc. Relat. Phenom.* **2007**, *155*, 47–53.

(17) Pavlychev, A. A.; Hallmeier, K. H.; Hennig, C.; Hennig, L.; Szargan, R. Nitrogen K-shell excitations in complex molecules and polypyrrole. *Chem. Phys.* **1995**, *201*, 547–555.

(18) Duflot, D.; Flament, J.-P.; Giuliani, A.; Heinesch, J.; Hubin-Franskin, M.-J. Core shell excitation of furan at the O1s and C1s edges: An experimental and ab initio study. *J. Chem. Phys.* **2003**, *119*, 8946–8955.

(19) Hitchcock, A. P.; Brion, C. E. K-shell excitation of HF and $F_2$ studied by electron energy-loss spectroscopy. *J. Phys. B: At. Mol. Phys.* **1981**, *14*, 4399.

(20) Barrie, A.; Drummond, I. W.; Herd, Q. C. Correlation of calculated and measured 2p spin–orbit splitting by electron spectroscopy using monochromatic x-radiation. *J. Electron Spectrosc. Relat. Phenom.* **1974**, *5*, 217–225.

(21) Fronzoni, G.; Stener, M.; Decleva, P.; Simone, M. d.; Coreno, M.; Franceschi, P.; Furlani, C.; Prince, K. C. X-ray Absorption Spectroscopy of $VOCl_3$, $CrO_2Cl_2$, and $MnO_3Cl$: An Experimental and Theoretical Study. *J. Phys. Chem. A* **2009**, *113*, 2914–2925.

(22) George, S. J.; Drury, O. B.; Fu, J.; Friedrich, S.; Doonan, C. J.; George, G. N.; White, J. M.; Young, C. G.; Cramer, S. P. Molybdenum X-ray absorption edges from 200 to 20,000 eV: The benefits of soft X-ray spectroscopy for chemical speciation. *J. Inorg. Biochem.* **2009**, *103*, 157–167.

(23) Boysen, R. B.; Szilagyi, R. K. Development of palladium L-edge X-ray absorption spectroscopy and its application for chloropalladium complexes. *Inorg. Chim. Acta* **2008**, *361*, 1047–1058.

(24) Jayarathne, U.; Chandrasekaran, P.; Greene, A. F.; Mague, J. T.; DeBeer, S.; Lancaster, K. M.; Sproules, S.; Donahue, J. P. X-ray Absorption Spectroscopy Systematics at the Tungsten L-Edge. *Inorg. Chem.* **2014**, *53*, 8230–8241.

(25) Konecny, L.; Komorovsky, S.; Vicha, J.; Ruud, K.; Repisky, M. Exact Two-Component TDDFT with Simple Two-Electron Picture-Change Corrections: X-ray Absorption Spectra Near L- and M-Edges of Four-Component Quality at Two-Component Cost. *J. Phys. Chem. A* **2023**, *127*, 1360–1376.

(26) Hayes, W.; Brown, F. C. Absorption by Some Molecular Gases in the Extreme Ultraviolet. *Phys. Rev. A* **1972**, *6*, 21–30.

(27) Bozek, J. D.; Tan, K. H.; Bancroft, G. M.; Tse, J. S. High resolution gas phase photoabsorption spectra of $SiCl_4$ and $Si(CH_3)_4$ at the silicon L edges: Characterization and assignment of resonances. *Chem. Phys. Lett.* **1987**, *138*, 33–42.

(28) Sutherland, D. G. J.; Kasrai, M.; Bancroft, G. M.; Liu, Z. F.; Tan, K. H. Si L- and K-edge x-ray-absorption near-edge spectroscopy of gas-phase $Si(CH_3)_x(OCH_3)_{4-x}$: Models for solid-state analogs. *Phys. Rev. B* **1993**, *48*, 14989–15001.

(29) Friedrich, H.; Sonntag, B.; Pittel, B.; Rabe, P.; Schwarz, W. H. E. Overlapping core to valence and core to Rydberg transitions and resonances in the XUV spectra of $SiF_4$. *J. Phys. B: At. Mol. Phys.* **1980**, *13*, 25.

(30) Liu, Z. F.; Cutler, J. N.; Bancroft, G. M.; Tan, K. H.; Cavell, R. G.; Tse, J. S. High resolution gas phase photoabsorption spectra and multiple-scattering X$\alpha$ study of $PX_3$ (X = H, $CH_3$, $CF_3$) compounds at the P $L_{2,3}$ edge. *Chem. Phys. Lett.* **1990**, *172*, 421–429.

(31) Neville, J. J.; Jürgensen, A.; Cavell, R. G.; Kosugi, N.; Hitchcock, A. P. Inner-shell excitation of $PF_3$, $PCl_3$, $PCl_2CF_3$, $OPF_3$ and $SPF_3$: Part I. Spectroscopy. *Chem. Phys.* **1998**, *238*, 201–220.

(32) Hu, Y. F.; Zuin, L.; Püttner, R. High-resolution gas phase P L-edge photoabsorption spectra of $PF_5$. *Can. J. Chem.* **2007**, *85*, 690–694.

(33) Hedin, L.; Eland, J. H. D.; Karlsson, L.; Feifel, R. An x-ray absorption and a normal Auger study of the fine structure in the $S2p^{-1}$ region of the $CS_2$ molecule. *J. Phys. B: At., Mol. Opt. Phys.* **2009**, *42*, 085102.

(34) Schnorr, K.; Bhattacherjee, A.; Oosterbaan, K. J.; Delcey, M. G.; Yang, Z.; Xue, T.; Attar, A. R.; Chatterley, A. S.; Head-Gordon, M.; Leone, S. R.; Gessner, O. Tracing the 267 nm-Induced Radical Formation in Dimethyl Disulfide Using Time-Resolved X-ray Absorption Spectroscopy. *J. Phys. Chem. Lett.* **2019**, *10*, 1382–1387.

(35) Ankerhold, U.; Esser, B.; von Busch, F. Ionization and fragmentation of OCS and $CS_2$ after photoexcitation around the sulfur 2p edge. *Chem. Phys.* **1997**, *220*, 393–407.

(36) Guillemin, R.; Stolte, W. C.; Dang, L. T. N.; Yu, S.-W.; Lindle, D. W. Fragmentation dynamics of $H_2S$ following S 2p photoexcitation. *J. Chem. Phys.* **2005**, *122*, 094318.

(37) Hudson, E.; Shirley, D. A.; Domke, M.; Remmers, G.; Puschmann, A.; Mandel, T.; Xue, C.; Kaindl, G. High-resolution measurements of near-edge resonances in the core-level photoionization spectra of $SF_6$. *Phys. Rev. A* **1993**, *47*, 361–373.

(38) Nayandin, O.; Kukk, E.; Wills, A. A.; Langer, B.; Bozek, J. D.; Canton-Rogan, S.; Wiedenhoeft, M.; Cubaynes, D.; Berrah, N. Angle-resolved two-dimensional mapping of electron emission from the inner-shell 2p excitations in $Cl_2$. *Phys. Rev. A* **2001**, *63*, 062719.

(39) Lu, K. T.; Chen, J. M.; Lee, J. M.; Chen, C. K.; Chou, T. L.; Chen, H. C. State-specific dissociation enhancement of ionic and excited neutral photofragments of gaseous $CCl_4$ and solid-state analogs following Cl 2p core-level excitation. *New J. Phys.* **2008**, *10*, 053009.

(40) Aksela, H.; Aksela, S.; Ala-Korpela, M.; Sairanen, O.-P.; Hotokka, M.; Bancroft, G. M.; Tan, K. H.; Tulkki, J. Decay channels of core-excited HCl. *Phys. Rev. A* **1990**, *41*, 6000–6005.

(41) Hitchcock, A. P.; Pocock, M.; Brion, C. E.; Banna, M. S.; Frost, D. C.; McDowell, C. A.; Wallbank, B. Inner shell excitation and ionization of the monohalobenzenes. *J. Electron Spectrosc. Relat. Phenom.* **1978**, *13*, 345–360.

(42) Sze, K. H.; Brion, C. E. Inner shell and valence shell electronic excitation of $ClF_3$ by high energy electron impact. An investigation of potential barrier effects. *Chem. Phys.* **1989**, *137*, 353–367.

(43) Tougerti, A.; Cristol, S.; Berrier, E.; Briois, V.; La Fontaine, C.; Villain, F.; Joly, Y. XANES

study of rhenium oxide compounds at the $L_1$ and $L_3$ absorption edges. *Phys. Rev. B* **2012**, *85*, 125136.

(44) Butorin, S. M.; Modin, A.; Vegelius, J. R.; Kvashnina, K. O.; Shuh, D. K. Probing Chemical Bonding in Uranium Dioxide by Means of High-Resolution X-ray Absorption Spectroscopy. *J. Phys. Chem. C* **2016**, *120*, 29397–29404.
